# The genomic landscape of Mongolian hepatocellular carcinoma

**DOI:** 10.1038/s41467-020-18186-1

**Published:** 2020-09-01

**Authors:** Julián Candia, Enkhjargal Bayarsaikhan, Mayank Tandon, Anuradha Budhu, Marshonna Forgues, Lkhagva-Ochir Tovuu, Undarmaa Tudev, Justin Lack, Ann Chao, Jigjidsuren Chinburen, Xin Wei Wang

**Affiliations:** 1 Laboratory of Human Carcinogenesis, Center for Cancer Research, National Cancer Institute, National Institutes of Health, Bethesda, MD 20892 USA; 2 General Laboratory Department, National Cancer Center, Ulaanbaatar, Mongolia; 3 CCR Collaborative Bioinformatics Resource, Center for Cancer Research, National Cancer Institute, National Institutes of Health, Bethesda, MD 20892 USA; 4 Liver Cancer Program, Center for Cancer Research, National Cancer Institute, National Institutes of Health, Bethesda, MD 20892 USA; 5 Cancer Registry and Screening Department, National Cancer Center, Ulaanbaatar, Mongolia; 6 Center for Global Health, National Cancer Institute, National Institutes of Health, Rockville, MD 20850 USA; 7 Hepato-Pancreatic-Biliary Surgical Department, National Cancer Center, Ulaanbaatar, Mongolia

**Keywords:** Cancer genomics, Hepatocellular carcinoma, Gene expression profiling

## Abstract

Mongolia has the highest incidence of hepatocellular carcinoma (HCC) in the world, but its causative factors and underlying tumor biology remain unknown. Here, we describe molecular characteristics of HCC from 76 Mongolian patients by whole-exome and transcriptome sequencing. We present a comprehensive analysis of mutational signatures, driver genes, and molecular subtypes of Mongolian HCC compared to 373 HCC patients of different races and ethnicities and diverse etiologies. Mongolian HCC consists of prognostic molecular subtypes similar to those found in patients from other areas of Asia, Europe, and North America, as well as other unique subtypes, suggesting the presence of distinct etiologies linked to Mongolian patients. In addition to common driver mutations (TP53, CTNNB1) frequently found in pan-cancer analysis, Mongolian HCC exhibits unique drivers (most notably GTF2IRD2B, PNRC2, and SPTA1), the latter of which is associated with hepatitis D viral infection. These results suggest the existence of new molecular mechanisms at play in Mongolian hepatocarcinogenesis.

## Abstract (web summary)

Mongolia has the highest incidence of—and mortality from—hepatocellular carcinoma (HCC) in the world. Here, the authors examine the genomic and transcriptomic landscape of Mongolian HCC, uncover novel driver mutations, and suggest distinct disease etiologies.

## Introduction

Liver cancer is the second most common cause of cancer mortality worldwide, with more than 840,000 annual new cases and 780,000 annual deaths recorded globally in recent years^1^. Hepatocellular carcinoma, the predominant form of liver cancer, has several known risk factors, including chronic hepatitis B virus (HBV) and/or hepatitis C virus (HCV) infection, autoimmune hepatitis, diabetes mellitus, alcohol abuse, obesity, and several metabolic diseases^2,3^. Mongolia has the highest reported incidence of—and mortality from—HCC in the world, which is between three and seven times higher than that observed in other high-incidence populations, such as South Korea, Thailand, and China^4,5^. In Mongolia, where cancer is the second most common cause of death accounting for nearly a fifth of all deaths, HCC is the most prevalent cancer type accounting for ~40% of all cancers. Besides chronic infection with HBV and/or HCV, present in more than 90% of Mongolian HCC cases^6,7^, the etiology of Mongolian HCC may also be related to the extraordinarily high prevalence of hepatitis delta virus (HDV)^8,9^, which depends on HBV for its life cycle. Among HBV-infected Mongolian subjects, ~60% were found HDV-coinfected, compared to the ~5% global estimate^9^. Despite the daunting magnitude of this longstanding health crisis, the molecular landscape of Mongolian HCC has not yet been studied. Our work fills this gap with the first comprehensive and integrative genomic characterization of Mongolian HCC, aiming to identify robust molecular subclasses with underlying unique tumor biology, as well as driver features informative of the etiology and progression of the disease.

## Results

### Identification of molecular subtypes of Mongolian HCC

**Fig. 1:**
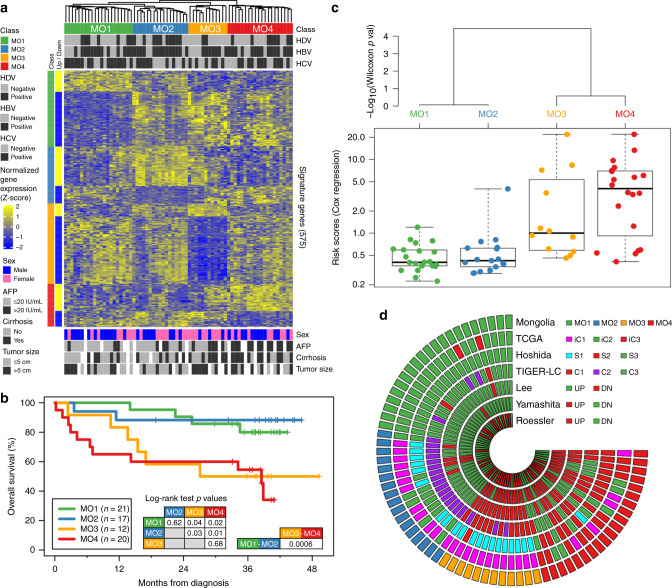
Transcriptome-based molecular subclasses of Mongolian hepatocellular carcinoma. **a** Heatmap showing normalized expression levels of 575 signature genes (rows) across subjects (columns) classified in four molecular subclasses, MO1-4, which were determined by an unsupervised approach (consensus clustering^10^). Each signature gene is significantly up- or down-regulated in one subclass relative to the other subclasses, as indicated by the annotation bars on the left side. Signature genes and subjects within each class were hierarchically clustered. Molecular subclasses and infection status of hepatitis virus HDV, HBV, and HCV across subjects are shown at the top. Significant demographic and clinical annotations are provided at the bottom. **b** Overall survival for subjects grouped according to molecular subclasses. Both individually and grouped pairwise, MO1-2 show statistically significant better prognosis compared with MO3-4 based on two-sided log-rank tests. **c** Main panel: risk scores, which were determined by a supervised approach (cross-validated regularized Cox regression^12^), grouped by subclass. Boxplots show median (thick horizontal line), first and third quartiles (lower and upper bounds of box, respectively), minimum and maximum (lower and upper whiskers, respectively). The number of biologically independent samples in each molecular subclass is *n* = 21 (MO1), 17 (MO2), 12 (MO3), and 20 (MO4). Top panel: Dendrogram based on two-sided Wilcoxon test *p* values between subclass pairs, which shows that MO1-2 and MO3-4 form different risk score groupings. **d** Classification of Mongolian subjects using different HCC gene signatures, displayed as different concentric rings: this study’s MO1-4 (outermost ring), followed inwards by TCGA^16^, Hoshida^17^, TIGER-LC^11^, Lee^18^, Yamashita^19^, and Roessler^20^ (innermost ring). Source data are provided as a Source Data file.

Clinical information and paired tumor/nontumor liver tissue samples were obtained with written informed consent from 76 HCC patients undergoing surgery between 2015 and 2016 at the National Cancer Center of Mongolia (Supplementary Data 1–2). Whole transcriptome sequencing and whole exome sequencing were performed on most tumor and adjacent nontumor tissues, followed by bioinformatics processing and quality control (Methods). Our transcriptomics-based analysis (Fig. 1) integrated an unsupervised approach (consensus clustering^10,11^ to uncover molecular subclasses) with a supervised approach (regularized Cox regression^12^ to find low- vs high-risk groups), followed by validation (mapping^13^ onto molecular subclasses from previous HCC studies). In order to uncover molecular subtypes of Mongolian HCC, we implemented an unsupervised clustering approach coupled with survival analysis (Supplementary Fig. 1) and found four molecular subclasses, labeled MO1-4 (Fig. 1a). Associated to these four molecular subclasses, we found 575 signature genes, each of them significantly up- or down-regulated in one subclass relative to the other subclasses (Supplementary Data 3). The strongest association observed between molecular subclasses and demographic/clinical variables was alpha-fetoprotein (AFP), primarily driven by opposite trends in MO4 (odds ratio [OR] = inf, *p* = 4 × 10^−6^, positively associated with abnormal AFP) vs MO1 (OR = 0.2, *p* = 0.008, negatively associated with abnormal AFP). This phenomenon was mirrored, albeit with marginal significance, by cirrhosis, which correlates positively with MO4 (OR = 3, *p* = 0.08) and negatively with MO1 (OR = 0.3, *p* = 0.08). Tumor size, which correlates positively with MO3 (OR = 8.9, *p* = 0.05), appeared as marginally significant (Supplementary Fig. 2). AFP is a well-characterized biomarker for diagnosis, pathological grade, progression, and survival of HCC patients^14^, whereas cirrhosis, on the other hand, is a well-known intermediate stage in the progression from chronic liver disease and fibrosis to liver tumorigenesis^15^. In agreement with these findings, Kaplan–Meier plots of overall survival showed that subclasses MO1-2 correspond to statistically significant better prognosis compared with MO3-4 (Fig. 1b). It is important to emphasize that each of these four molecular subclasses is characterized by a unique transcriptomic profile with distinctive differentially expressed pathways (Supplementary Fig. 3a and Supplementary Data 4). Furthermore, paired tumor-vs-nontumor comparisons reveal a large number of differentially expressed genes in each molecular subclass, many of which are shared among two or more subclasses (Supplementary Fig. 3b-c and Supplementary Data 5). It is interesting to notice, however, that MO2 appears to have an order of magnitude fewer tumor-vs-nontumor differentially expressed genes compared to the other subclasses; correspondingly, MO2 will be shown to carry fewer copy number variations (CNVs) and structural variants (SVs) (see below). By implementing a regularized Cox regression approach (Supplementary Fig. 4 and Supplementary Data 6), we found well defined low- and high-risk groups (*p* = 5 × 10^−10^). The risk scores are in good agreement with the molecular subclasses defined earlier (Fig. 1c) and confirm the association of MO1-2 with better prognosis/low-risk and that of MO3-4 with poorer prognosis/high-risk. Risk scores also highlight the existence of outcome-associated heterogeneities within transcriptome-derived molecular subclasses, most notably within MO3 and MO4. For the interpretation of results in the remainder of this study, we keep track of modular subclasses and risk categories as informative subcohort stratification signatures. To validate these findings, we compared the classification of Mongolian patients across subclass-related gene signatures from different studies (Fig. 1d). Signatures are represented as concentric rings, starting with this study’s MO1-4 (outermost ring), followed inwards by TCGA^16^, Hoshida^17^, TIGER-LC^11^, Lee^18^, Yamashita^19^, and Roessler^20^ (innermost ring). Transcriptomics-based gene signatures, either available from the Molecular Signatures Database^21^ or inferred from gene expression data (“Methods”), are provided in Supplementary Data 7. The overlap between signature genes in each of these HCC studies and signature genes in Mongolian HCC is not significant at the *p* = 0.05 threshold level based on Fisher’s exact test. The association between the molecular subclasses from this study, MO1-4, and those from each one of the previous studies considered here, is statistically significant (Fisher’s exact test *p* value < 0.05) (Supplementary Fig. 5). Subclasses MO1 and MO4 appear mostly stable and consistent across studies, while MO2 and MO3 appear more heterogeneous. It is worth noticing that most of the gene signatures from previous HCC studies appear to have informative prognostic value when applied to Mongolian HCC survival (Supplementary Fig. 6). Comparing prognostic prediction performance in a cross-validated framework, however, confirms that this study’s signature is the most informative to predict survival in Mongolian HCC, as would be expected (Supplementary Fig. 7). TCGA^16^ reports the existence of an IDH-like transcriptome phenotype associated with the poor prognosis iC1 subclass. Whereas none of the Mongolian HCC tumor samples was found to carry mutations in IDH1 or IDH2, we observed a subset of 9 samples in the Mongolian cohort that appears to carry TCGA’s IDH-like gene signature (Supplementary Fig. 8a). Of these, 3 belong to MO2 and 6 to MO3 (Supplementary Fig. 8b), which agrees with the fact that subclasses MO2-3 are strongly associated with TCGA’s subclass iC1 (see Fig. 1d above); no significant associations between IDH-like status and demographic/clinical variables were found. In agreement with TCGA’s observations, IDH-like samples appear associated with poorer prognosis (Supplementary Fig. 8c).

### Somatic drivers of Mongolian HCC

**Fig. 2:**
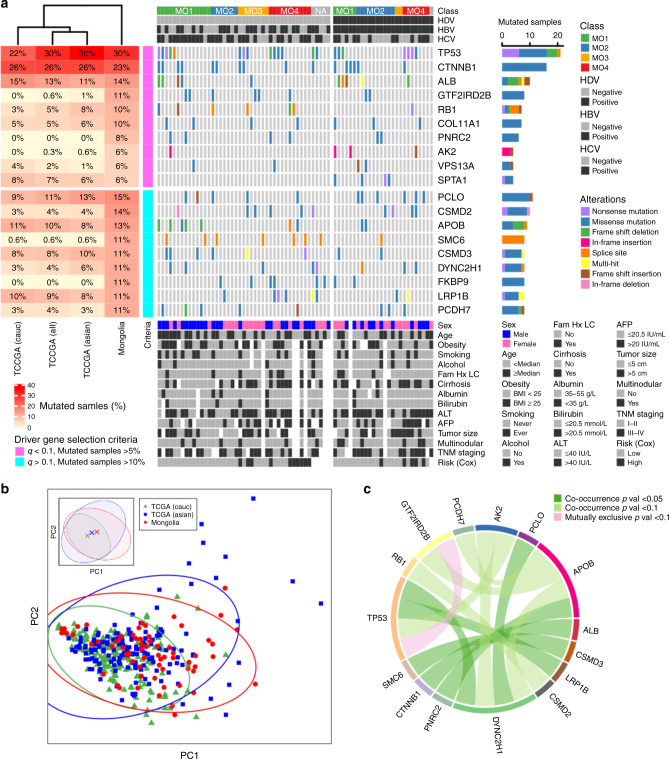
Mutational landscape of Mongolian hepatocellular carcinoma. **a** Oncoplot showing mutated driver genes (rows) across subjects (columns) split by HDV status (left panel: HDV-; right panel: HDV+). The top panel shows 10 genes selected by the combined criteria of MutSigCV *q* value < 0.1 and fraction of mutated samples >5%, while the bottom panel shows 9 additional genes that appear frequently mutated (>10% of samples). The left-side table shows the fraction of mutated samples in the Mongolian cohort compared to TCGA-LIHC (full cohort and split by major racial subgroups, namely Asian and Caucasian). Molecular subclasses and infection status of hepatitis virus HDV, HBV, and HCV across subjects are shown at the top. Demographic and clinical annotations are provided at the bottom, as well as risk groups based on this study’s supervised transcriptome analysis. **b** Principal component analysis of somatic substitution patterns in the Mongolian cohort compared to those from TCGA-LIHC’s major racial subgroups. Symbols represent individual patients. Also shown are the 95%-CL ellipses corresponding to each cohort. Inset: ellipses and centroids for each cohort. **c** Ribbon plot showing the co-occurrence (or mutually exclusive relation) between pairs of mutated driver genes in the Mongolian cohort. Source data are provided as a Source Data file.

We determined the mutational landscape of Mongolian HCC (Fig. 2) compared to previous studies of driver mutations in 373 HCC patients from different races and ethnicities, as well as from geographic locations with varying etiologies^16^. The median mutation burden was 2.12 mutations/Mb and distributed similarly to TCGA-LIHC across variant subtypes (Supplementary Fig. 9). The oncoplot (Fig. 2a) shows mutated driver genes across the cohort split into two panels by HDV status due to the uniquely high prevalence of HDV in Mongolian HCC; within each panel, subjects are ordered by transcriptome-based molecular subclass. Demographic and clinical characteristics are also included for comparison. The top panel shows 10 genes selected by the criteria of MutSigCV^22^ *q* value < 0.1 and fraction of mutated samples >5%; the bottom panel shows 9 additional genes that, despite larger *q* values, appear mutated in more than 10% of the samples (Supplementary Data 8). The table on the left shows the fraction of mutated samples for the Mongolian cohort compared to TCGA-LIHC (full cohort and split by the two main racial subgroups, namely Asian and Caucasian). A further comparison of Mongolian HCC with TCGA-LIHC racial subgroups is shown as a principal component analysis (PCA) of somatic substitution patterns^23^ (Fig. 2b). For this analysis, each subject was first represented by the normalized mutational frequency along 96 trinucleotides, formed by enumerating all single-nucleotide combinations before and after each one of 6 possible single-nucleotide substitutions^24,25^. In anticipation of a more detailed analysis of mutational spectra and their connection to annotated etiologies from known signature catalogs (presented below), here PCA offers a straightforward low-dimensional representation to visualize the somatic mutational burden of many subjects across different cohorts. More specifically, we use PCA to focus on the 2 orthogonal directions of largest variance, PC1 and PC2, in order to uncover overall characteristics of Mongolian HCC compared with TCGA-LIHC’s Asian and Caucasian groups. Mutational frequency patterns are linked to mutation-causing mechanisms at the molecular level, as well as to disease etiologies at the organismal level. Therefore, the relative centroid location and spread of different cohorts provides a high-level view of their relative overall similarity and of their comparative mutational and etiological heterogeneity, respectively. In this representation, the Mongolian and Asian TCGA cohorts display larger spreads than the Caucasian TCGA cohort. The inset shows the centroids of each cohort, where it becomes apparent the Caucasian TCGA’s shift along both PC axes (Supplementary Data 9). Out of 19 candidate driver genes identified in our analysis, 8 of them have been reported as driver genes in at least one of 12 previous HCC studies^11,16,23,26–34^, while 11 genes (GTF2IRD2B, PNRC2, AK2, VPS13A, SPTA1, PCLO, CSMD2, SMC6, DYNC2H1, FKBP9, and PCDH7) have not been reported before (Supplementary Data 10). Among them, SPTA1, which encodes α-spectrin, displays mutations significantly associated with HDV + (*p* = 0.015). SPTA1 mutations have been linked to hereditary elliptocytosis and hereditary spherocytosis, a set of congenital hemolytic syndromes^35^. Although not reported as driver gene in previous HCC studies, SPTA1 was reported as a possible tumor suppressor in Glioblastoma Multiforme^36^. However, it is unclear how mechanistically α-spectrin contributes to tumorigenesis. GTF2IRD2B belongs to the TFII-I family of general transcription factors that play a role in chromatin structure modification and, consequently, in the regulation of gene expression^37^. It is plausible that mutations of GTF2IRD2B may lead to the disruption of gene expression regulation, thereby contributing to HCC carcinogenesis. In contrast, a number of genes found to be significantly mutated in other HCC cohorts (most notably AXIN1, ARID1A, ARID2, RPS6KA3, NFE2L2, and TERT, reported in at least half of the previous HCC studies here considered) do not appear significantly mutated in Mongolian HCC (Supplementary Data 11). These results are consistent with the hypothesis of the existence of new molecular mechanisms at play in Mongolian hepatocarcinogenesis. In order to gain further insight, Fig. 2c shows the significant mutation co-occurrence of Mongolian driver genes across the cohort. The incidence of mutations in TP53 and GTF2IRD2B, however, shows a case of significant mutual exclusivity. Although the analysis of co-occurrence in subcohorts suffers from weak statistical power, we found significant associations between pairs of driver genes in molecular subclasses, both individually and grouped by survival outcome (Supplementary Fig. 10 and Supplementary Data 12), which is consistent with the hypothesis that a combination of activation of multiple oncogenes and/or tumor suppressor genes may be needed to drive hepatocarcinogenesis. While we found different frequencies of driver mutations in HDV-associated HCC, the oncogenic roles of HDV in HCC could only be speculated^38^. Taken together, these analyses suggest the existence of unique driver mutations linked to Mongolian HCC. Further studies on additional Mongolian HCC specimens may be needed to validate these findings.

**Fig. 3:**
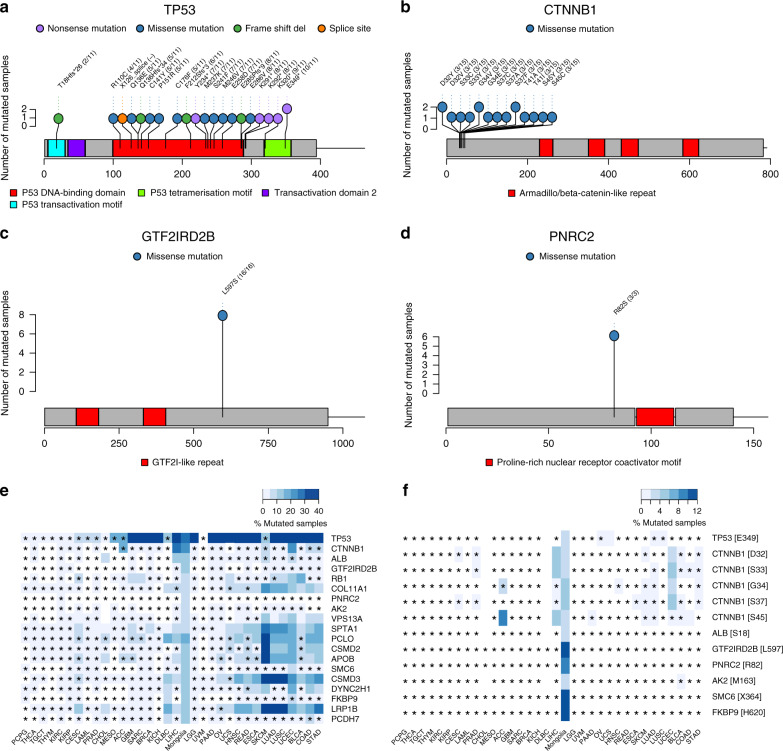
Hotspot mutations in Mongolian hepatocellular carcinoma. Mutated loci are shown for (**a**) TP53, (**b**) CTNNB1, (**c**) GTF2IRD2B, and (**d**) PNRC2. The number of mutated samples at each locus is displayed. In parentheses, exon numbers are also shown. **e** Frequency of mutated samples for candidate driver genes in the Mongolian cohort compared against all other cancer types available from The Cancer Genome Atlas (TCGA). Statistically significant differences in mutation frequencies from the Mongolian cohort are indicated by asterisks (one-tailed hypergeometric test without multiple-testing correction, *p* < 0.05). **f** Frequency of mutated samples for hotspots (defined as candidate driver gene loci mutated in two or more samples in the Mongolian cohort) compared against all other TCGA cancer types. Statistically significant differences in mutation frequencies from the Mongolian cohort are indicated by asterisks (one-tailed hypergeometric test without multiple-testing correction, *p* < 0.05). Source data are provided as a Source Data file.

While the location of mutations for the two most frequently mutated genes, TP53 and CTNNB1, was consistent with previously published studies (Fig. 3a, b), two novel driver genes, GTF2IRD2B and PNRC2, notably display hotspot missense mutations (Fig. 3c, d). Interestingly, while a majority of TP53 mutations were located in the DNA binding domain, we found two cases with E349 mutations, a locus in the p53 tetramerization domain known to affect p53 transcriptional activity^39^. GTF2IRD2B has the L597S allele in all 8 mutated cases while PNRC2 has the R82S allele in all 6 mutated cases. Supplementary Data 13 contains detailed information of all mutated loci among Mongolian HCC driver genes, including the predicted variant pathogenicity from ClinVar^40^, SIFT^41^, and PolyPhen^42^. Figure 3e displays the mutation frequency of these genes across all TCGA cancer studies. GTF2IRD2B and PNRC2 appear to carry hotspot mutations unique to Mongolian HCC; their mutation frequency is significantly higher than that observed in any other cancer type, as indicated by the asterisks. In contrast, other genes, such as SPTA1, which we found to be associated with HDV, appear significantly mutated in multiple other cancer types. Rather than summarizing per gene, Fig. 3f shows a pan-cancer comparison of mutation frequency for each hotspot locus. These hotspot loci are more frequently mutated in Mongolian HCC than in most other cancer types.

### Mutational signatures of Mongolian HCC

**Fig. 4:**
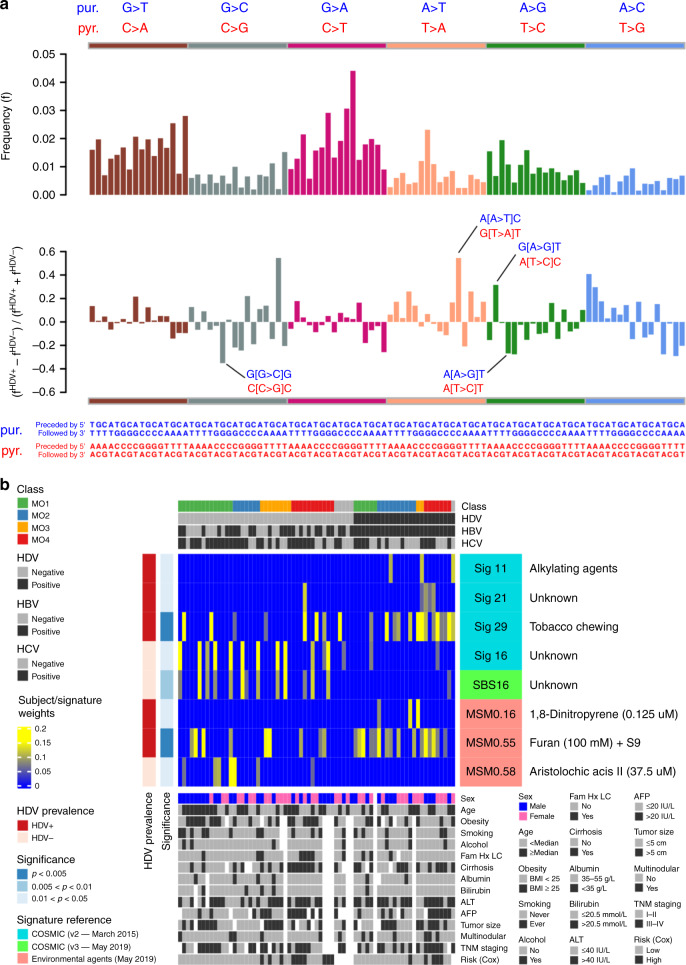
Mutational signatures of Mongolian hepatocellular carcinoma. **a** Top panel: Mutational trinucleotide frequency distribution in the Mongolian cohort. For each one of 6 possible single-nucleotide substitutions (annotated at the top and shown in different colors), there correspond 16 combinations of preceding (5′ end) and following (3′ end) nucleotides (annotated at the bottom). Due to strand complementarity, two equivalent sets of annotations are possible, either based on the substitution of purines (blue) or pyrimidines (red). Bottom panel: Differential frequency distribution in HDV+ patients relative to HDV-. Significant differences in substitution frequencies are indicated. **b** Heatmap showing subject/signature weights obtained from non-negative least squares mapping^43^ of individual samples (columns) vs reference signatures (rows) from the COSMIC catalogs^24,25^ and the Compendium of Mutational Signatures of Environmental Agents^44^, which identifies signatures with distinct prevalence among HDV + and HVD- groups (two-sided Wilcoxon test, *p* < 0.05). Molecular subclasses and infection status of hepatitis virus HDV, HBV, and HCV across subjects are shown at the top. Demographic and clinical annotations are provided at the bottom, as well as risk groups based on this study’s supervised transcriptome analysis. Source data are provided as a Source Data file.

To explore the etiology of Mongolian HCC, we analyzed mutational signatures that consist of frequency patterns along 96 trinucleotides, formed by enumerating all single-nucleotide combinations before and after each one of 6 possible single-nucleotide substitutions^24,25^. Figure 4a shows the frequency distribution of single-nucleotide substitutions in the Mongolian cohort (top) and the differential frequency distribution in HDV^+^ patients relative to HDV^-^ (bottom). By comparing the observed HDV± differences to a null model distribution obtained from random permutations of sample labels, significant differences (*p* < 0.05) are observed in A[A > T]C (G[T > A]T) and G[A > G]T (A[T > C]C) substitutions, which appear in excess in HDV^+^ tumors, as well as G[G > C]G (C[C > G]C) and A[A > G]T (A[T > C]T) substitutions, which appear in excess in HDV^-^ tumors (Supplementary Data 14). Figure 4b shows a heatmap of subject/signature weights from non-negative least squares mapping^43^ of individual samples vs reference signatures^24,25,44^, which identifies signatures with distinct prevalence among HDV+ and HDV− groups. Signatures differentially associated with HDV+ include mutational patterns linked to alkylating agents (such as temozolomide), tobacco chewing, and exposures to 1,8-Dinitropyrene and furan, whereas HDV- appears differentially associated with aristolochic acid II. Tobacco smoking and HBV infection were found to positively interact in liver carcinogenesis;^45,46^ synergistic effects were also reported between tobacco smoking and chronic HCV leading to increased liver fibrosis^46,47^ and increased risk of cirrhosis and HCC^46^. Exposure to aristolochic acids, widely used in traditional Chinese medicine throughout Asia, has been linked to liver cancer^48^, and a prospective study of HCV-infected patients in Taiwan has very recently reported an association between the intake of herbal medicines containing aristolochic acid and the risk of primary liver cancer^49^. Therefore, although the etiology of this disease and the role of HDV remain poorly understood, our findings suggest links between exposure to certain carcinogenic agents and HDV status in Mongolian HCC that deserve further investigation.

### Copy number alterations, gene fusions, structural variants, and germline analyses of Mongolian HCC

**Fig. 5:**
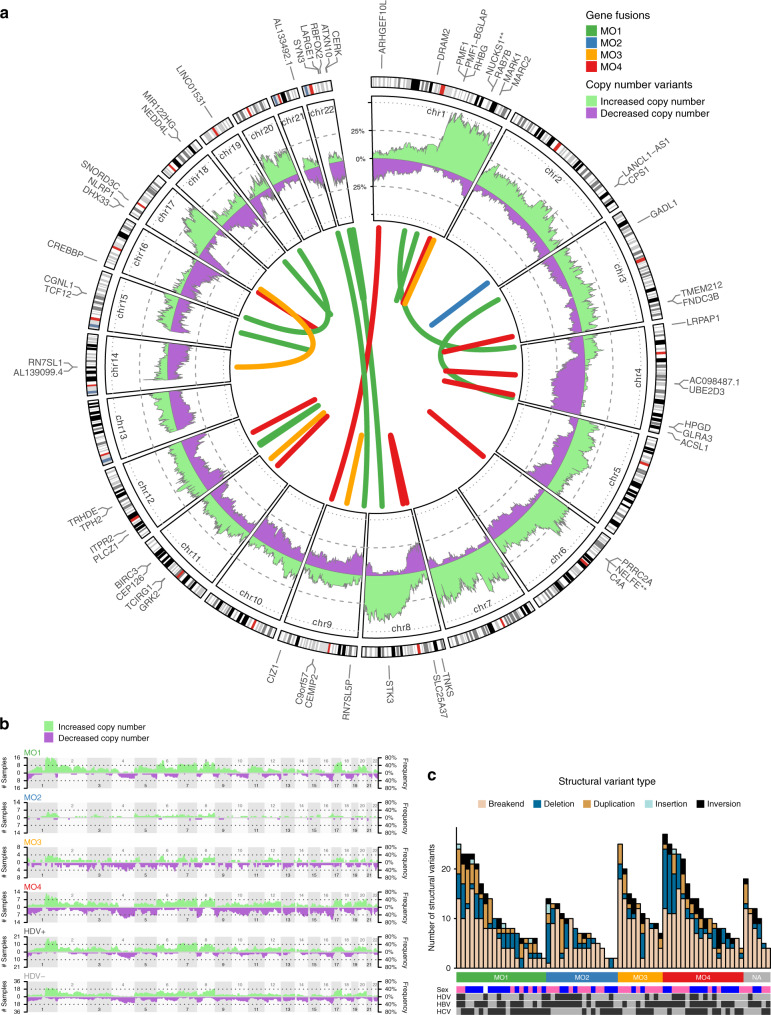
Copy number alterations, gene fusions, and structural variants in Mongolian HCC. **a** Integrated view showing the percent of samples with aberrant DNA copy number gains (green) and losses (purple) along the exome (inner ring) and gene fusions colored by molecular subclass (inner circle links). The autosome map and fused genes’ labels are provided as reference (outer ring). **b** Number of samples (left-side axis) and percent of samples (right-side axis) with aberrant DNA copy number gains (green) and losses (purple) along the exome separately shown for each molecular subclass and HDV± subcohorts. **c** Number of structural variants per subject. Source data are provided as a Source Data file.

Chromosomal abnormality is a hallmark of solid malignancies^50^. Indeed, ~90% of solid tumors are aneuploid, ranging from 26% in some tumor types to 99% in others^51^. In addition to point mutations, our data allow us to characterize other types of genomic aberrations, such as gene fusions, CNVs, SVs, and germline analyses. The inner circle in Fig. 5a shows gene fusions detected in individual samples, whose molecular subclass is indicated by link color. As reference, the fused genes’ labels are shown along the autosome map in the outer ring. (Supplementary Data 15). Interestingly, we found several fusion genes in one MO4 subject involving NELFE, an oncogene known to promote HCC progression via activation of myc signaling^52^. These results suggest that NELFE activation may contribute to hepatocarcinogenesis in Mongolian HCC. The inner ring in Fig. 5a displays the percent of samples in the Mongolian HCC cohort with CNVs across all autosomal chromosomes (Supplementary Data 16). In good agreement with overall CNV features in previous HCC studies^16,53^, we observe very significant gains in chromosome regions 1q and 8q, as well as significant losses in 1p, 4q, and 8p. Figure 5b shows the number (left-side axis) and percent (right-side axis) of samples affected by CNVs in each molecular subclass and HDV± subcohorts. Although following the overall patterns described above, MO2 displays a remarkably lesser impact of CNVs compared to MO1, despite both being associated with better outcome. Although to a lesser extent, we also observe MO3 less affected by CNVs compared to MO4. The distribution of SVs per subject, molecular subclass, and structural variant type is shown in Fig. 5c (Supplementary Data 17), which emphasizes again MO2 as generally less affected by these genomic aberrations compared with the other molecular subclasses. Furthermore, two types of germline analyses were performed. Germline-based sample admixture and similarity results are shown in Supplementary Fig. 11 for all tumor and adjacent nontumor samples. On the one hand, we observe that all matched tumor/nontumor pairs cluster together with the highest degree of similarity, thus providing an additional layer of quality control of our data. On the other hand, all samples appear classified as >99% East-Asian according to the 1000 Genomes Super Populations, which confirms the racial make-up expected of a Mongolian cohort. Finally, similarity clusters do not appear strongly correlated with molecular subclass labels, thus, suggesting that tumor molecular subclasses may be weakly dependent on germline characteristics. It should be noted that pairwise relatedness between samples in the cohort (used to generate the circular dendrogram in Supplementary Fig. 11) was computed using 17,766 common variants across all human populations. Genetic distance based on all germline variants found in the Mongolian HCC cohort also failed to show any significant patterns of similarity among MO1-4 clusters (data not shown). Therefore, it is unlikely that the four Mongolian subclasses reported here have a basis in germline characteristics but instead represent different pathologies primarily driven by somatic processes. Germline predisposition variant analysis was also performed on a panel of known cancer-causing genes, which only yielded ClinVar variants annotated as benign, likely benign, or VUS (variants of unknown significance). No other coding, non-ClinVar variants were found among the predisposition genes. The aggregated Cancer Predisposition Sequencing Report is herewith provided as Supplementary Data 18.

### Mutated oncogenic signaling pathways of Mongolian HCC

**Fig. 6:**
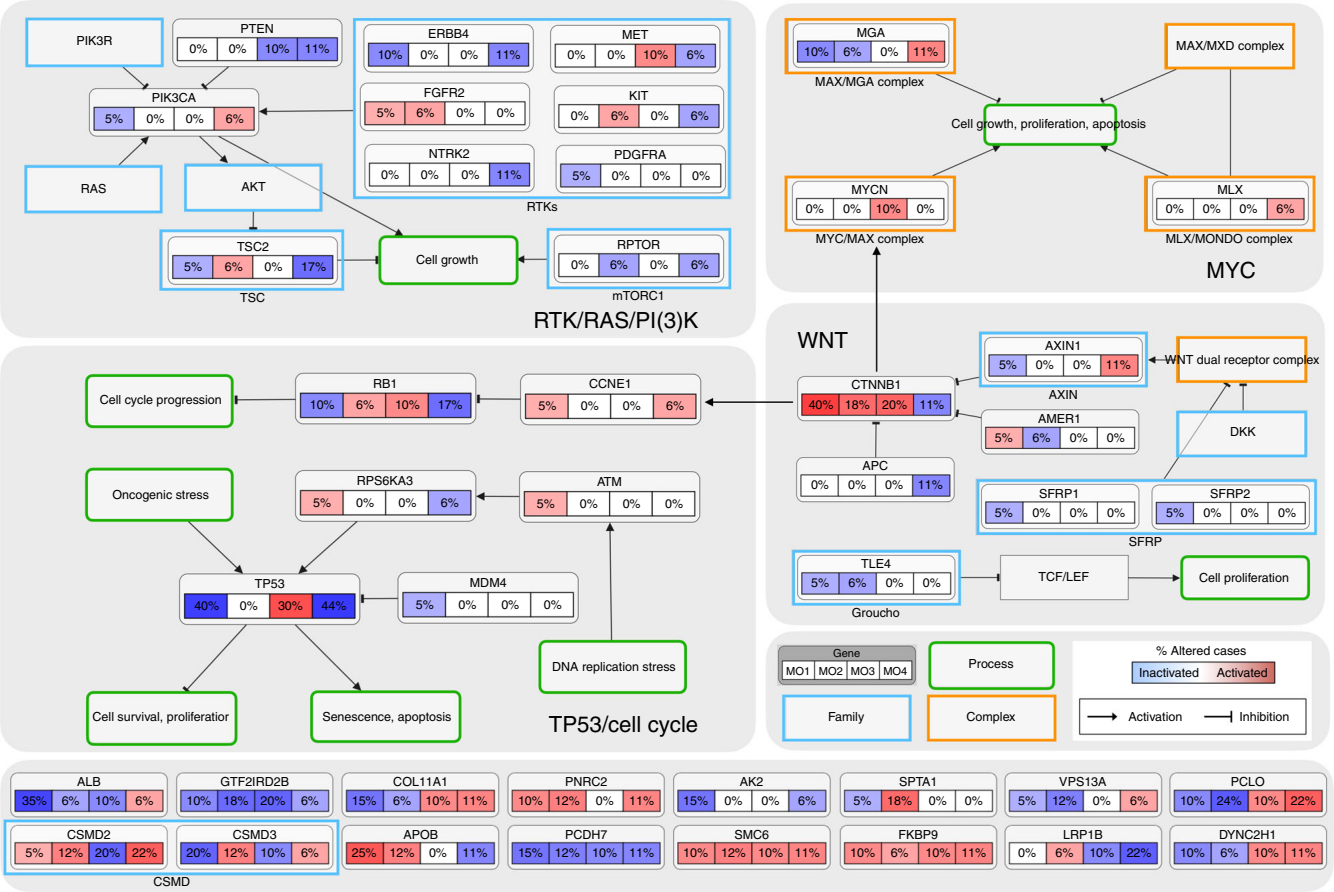
Mutated oncogenic signaling pathways and driver genes in the Mongolian cohort. Mutated genes and their activation/inhibition relationships to other genes, families, complexes, and cellular processes are displayed using curated pathway templates distilled from TCGA^54,95^. For each gene, the fraction of mutated samples within each molecular subclass, MO1-4, is shown. From the analysis of paired tumor-vs-nontumor gene expression ratios, the effect on each mutated gene (as activated or inactivated) within each molecular subclass is also provided. The bottom panel shows novel candidate driver genes in Mongolian hepatocellular carcinoma reported in this study.

In order to summarize the complex interplay of genomic alterations of Mongolian HCC, it is useful to represent them in the context of pathways associated with well-established hallmarks of cancer. Figure 6 shows the main mutated oncogenic signaling pathways^54^ and driver genes in the Mongolian cohort. As pointed out earlier, molecular subclasses are characterized by distinct up- and down-regulated pathways at the transcriptome level; correspondingly, each molecular subclass carries a distinct pattern of oncogenic signaling pathway alterations, which highlights the underlying molecular complexity of Mongolian HCC. For example, mutated TP53 has a different impact on tumor-vs-nontumor gene expression for molecular subclass MO3, in which tumor expression appears increased relative to nontumor expression, compared to MO1 and MO4, which exhibit the opposite trend. This ambivalent role of TP53 in tumorigenesis is well documented^55^ and consistent with our earlier study^56^ on the role of p53-mediated signaling in HCC. On the other hand, CTNNB1 mutations appear enriched in MO1 with good prognosis, which is consistent with TCGA data^16^. These results reaffirm several key signaling pathways commonly found during hepatocarcinogenesis, as shown in previous studies^11,16,23,26–34^. Remarkably, we also found several unique driver genes (bottom panel in Fig. 6), whose functions have not been studied in human cancer yet, which may represent processes of molecular pathogenesis unique to the Mongolian population. Further studies are needed to understand mechanistically the roles of these genes in Mongolian HCC, which in turn may inform better treatment strategies.

In summary, this study reports the landscape of driver genes, molecular subtypes, and associated tumor biology in Mongolian HCC, a tumor type with an unusually high prevalence in select geographic and demographic populations. We identified several unique driver genes, namely GTF2IRD2B, PNRC2, AK2, VPS13A, SPTA1, PCLO, CSMD2, SMC6, DYNC2H1, FKBP9, and PCDH7, that have not previously been reported, as well as complex mutation signatures linked to Mongolian liver tumors. Our results highlight the existence of novel molecular mechanisms at play in Mongolian hepatocarcinogenesis. Investigation of the functional roles and potential targeting of these driver genes in larger cohorts are warranted to help improve precision oncology and overcome the pressing liver cancer health crisis in Mongolia.

## Methods

### Liver samples and clinical data

HCC patients were diagnosed via standardized pathology reviews based on the WHO Classification of Tumors (also known as the WHO Blue Books) and via clinical assessments based on CT scans and ultrasound diagnosis. Tumoral and adjacent nontumoral liver tissue samples were collected and frozen at −80 °C after surgical resection at the National Cancer Center in Ulaanbaatar, Mongolia. The study was approved by the Ethics Committee at the National Cancer Center in Ulaanbaatar, Mongolia, and written informed consent was obtained from all participants. Cohort details are provided in Supplementary Data 1-2.

### Sequencing datasets

Out of 76 subjects in the Mongolian HCC cohort, we obtained paired tumor/nontumor datasets from total RNA sequencing and whole exome sequencing for 65 subjects. For 5 subjects, only total RNA sequencing data were generated for downstream analysis. Similarly, for 6 subjects, only whole-exome sequencing data were generated for downstream analysis.

### Total-RNA sequencing

RNA was extracted from 70 HCC tumors and paired nontumor liver tissues, for a total of 140 samples that were used for total RNA sequencing. Library preparation was performed using the Illumina TruSeq Stranded Total RNA Kit and sequenced across two flowcells on the Illumina NovaSeq platform, which yielded between 69 and 605 million reads per sample. The sequencing quality of the reads was assessed using FastQC (v. 0.11.5), Preseq^57^ (v. 2.0.3), Picard tools (v. 1.119), and RSeQC (v. 2.6.4). Reads were trimmed using Cutadapt^58^ (v. 1.14) to remove sequencing adapters prior to mapping to the human reference genome hg38 using STAR^59^ (v. 2.5.2b) in two-pass mode. Across the samples, the median percentage of mapped reads was 95.4%. Expression levels were quantified using RSEM^60^ (v. 1.3.0) with GENCODE^61^ annotation (v. 21). Genes with a mean count lower than one transcript were removed and the resulting data were normalized using the voom algorithm^62^ from the Limma R package^63^ (v. 3.40.6) for downstream analyses.

### Unsupervised clustering and survival analysis

In order to determine the optimal partition of the RNA-Seq-based cohort (*n* = 70) into biologically relevant molecular subclasses, we performed a grid-search-based clustering analysis coupled with survival analysis. Firstly, the most variable genes across tumor samples were selected using different median absolute deviation (MAD) thresholds (Supplementary Fig. 1a). The number of selected genes ranged from 9827 (for MAD threshold = 1) down to 522 (for MAD threshold = 3). Then, for each MAD threshold, we generated K-means-based consensus clustering solutions in the range *K* = 2–8 (where K represents the pre-assigned number of clusters). Using the R package ConsensusClusterPlus^10^ (v. 1.48.0), each of these solutions was generated from 1000 iterations, each iteration consisting of a randomized selection of 80% of samples and 80% of features (genes) to avoid overfitting. Each consensus clustering solution is summarized by the pairwise coclustering matrix, which captures the probability for two samples to be clustered together. By defining in = mean pairwise coclustering within a cluster and out = mean pairwise coclustering across clusters, the normalized ratio in/(in + out) was adopted as the objective function to find the optimal solution in a grid search across different MAD thresholds and different numbers of clusters (Supplementary Fig. 1b). The best solutions were the 2-cluster solution with MAD threshold = 1.25 (Supplementary Fig. 1c) and the 4-cluster solution with MAD threshold = 2 (Supplementary Fig. 1d). Kaplan–Meier and log-rank test survival analyses were performed, showing that the 2-cluster solution failed to capture prognosis-relevant information (Supplementary Fig. 1e). In contrast, the 4-cluster solution showed statistically significant survival differences across the clusters (Supplementary Fig. 1f) and was adopted to define molecular subclasses MO1-4. The association between the 2-cluster solution and the better (MO1-2)/worse (MO3-4) survival groups derived from the 4-cluster solution is not significant (OR = 0.86, Fisher’s exact test *p* value = 0.81).

### Significance tests of contingency tables

The association between two categorical variables, such as molecular subclasses and clinical variables (Supplementary Fig. 2) or molecular subclasses from classifications derived from other HCC studies (Supplementary Fig. 5), was assessed by Fisher’s exact test. When testing a 2 × 2 contingency table, we also report the OR, which can be expressed as the product of the diagonal entries divided by the product of the off-diagonal entries.

### Differential expression and pathway analysis

Differentially expressed genes associated with tumor samples in each molecular subclass were ranked by Wilcoxon test *p* value; the top 2000 genes were then uploaded to QIAGEN Ingenuity Pathway Analysis (v. 52912811) (Supplementary Fig. 3a and Supplementary Data 4). For tumor-vs-nontumor comparisons, differentially expressed genes within each molecular subclass were assessed by paired t-test and selected by FDR-adjusted *p* value < 0.05 (Supplementary Fig. 3b-c and Supplementary Data 5). The Venn diagram was generated with R package VennDiagram (v. 1.6.20). Since tumor and nontumor samples were separately run in two different batches, we checked the expression of reference housekeeping genes reported as stable across tumor and normal tissues^64–66^, including reference genes validated in paired tumoral and adjacent nontumoral tissues from HCC patients^66^ (Supplementary Fig. 12). This served as a further quality check of our data for the paired tumor-vs-nontumor analysis.

### Regularized Cox regression

As a preprocessing step, Reactome pathways^67^ were used to determine pathway-level expression using PC1, the first principal component. The resulting expression matrix of 2211 pathways and 5 key demographic and clinical control variables (sex, age, and HCV, HBV, and HDV status) was analyzed by means of a cross-validated elastic net implementation of regularized Cox regression using eNetXplorer^12^ (v. 1.1.0). Significant pathways, selected based on feature frequency (Supplementary Fig. 4a) and feature coefficient (Supplementary Fig. 4b) in the most stringent (lasso) solution, were used in a Cox regression model to determine risk scores and classify patients as low- vs high-risk (Supplementary Fig. 4c). Risk score stratification was validated by means of Kaplan–Meier and log-rank test survival analysis (Supplementary Fig. 4d).

### Mapping to HCC molecular subclass signatures

Subjects in the Mongolian cohort were mapped into molecular subclass signatures reported in other HCC studies (TCGA^16^, Hoshida^17^, TIGER-LC^11^, Lee^18^, Yamashita^19^, and Roessler^20^) using GenePattern’s NearestTemplatePrediction module^13,68^ (v. 4) and visualized with circlize^69^ (v. 0.4.8). For TCGA and TIGER-LC cohorts, molecular subclasses were originally obtained by iCluster^70^, an approach that merges molecular information from multiple sources. Based on the classification of individual subjects and their corresponding gene expression, we inferred transcriptomics-based signatures of up-regulated genes characteristic of each molecular subclass. For other HCC studies, transcriptomics-based signatures were readily available from the Molecular Signatures Database^21^ (v. 7.0). These gene signatures are provided in Supplementary Data 7. To compare prognostic performance across signatures in a cross-validated framework (Supplementary Fig. 7), we performed 200 runs with tenfold cross-validation. For each run and each fold, signature genes were used to build a lasso-regularized Cox regression model and predict risk on out-of-bag instances. Once all 10 folds were evaluated, concordance (also known as C-index) was measured to quantitatively compare out-of-bag predictions against the survival response. Packages used were survival (v. 3.1.8), survcomp^71^ (v. 1.34.0), and glmnet^72^ (v. 3.0.1).

### IDH-like gene signature analysis

In order to compare TCGA with Mongolian HCC samples, we applied the same data processing pipeline (voom normalization followed by *z*-score transformation) to both gene expression datasets. Only genes reported in both datasets were used. Following TCGA’s reported procedures^16^, the IDH-like gene signature was obtained by performing a *t* test comparison between IDH-mutant vs IDH-WT samples (*p* < 0.0001). To validate the approach, we used the gene signature to cluster TCGA samples hierarchically; IDH-mutant samples indeed formed a tight cluster, while IDH-like samples with score IDH_P > 0.8 were observed to form an adjacent cluster. By applying the hierarchical clustering procedure to the combined TCGA and Mongolian HCC datasets, we identified a group of 9 Mongolian samples that appeared to carry TCGA’s IDH-like signature (Supplementary Fig. 8a) followed by Kaplan–Meier and log-rank test survival analysis (Supplementary Fig. 8c).

### Gene fusion analysis

STAR-fusion^73^ (v. 1.6) was used for detecting fusion events in the RNA-Seq data for each sample. Events reported with FFPM <0.5 or a split read count <30 were removed from all samples. Fusion gene pairs detected in any normal sample were removed from each tumor sample to produce a set of high confidence driver fusion events. More information about the annotations supplied for each fusion event in Supplementary Data 15 can be found at https://github.com/FusionAnnotator/CTAT_HumanFusionLib/wiki

### Whole-exome sequencing

DNA was extracted from 72 HCC tumors and paired nontumor liver tissues, for a total of 144 samples that were used for library preparation using the Agilent SureSelect Human All Exon v7 exome capture kit and sequenced across three flowcells on the Illumina HiSeq 4000 Platform. Reads were trimmed for adapters and low-quality bases using Trimmomatic software before alignment to the human hg38 reference genome using BWA mapping software^74^ (v. 0.7.17). Mapped reads were then de-duplicated using Picard tools (v. 1.119), followed by re-alignment, and base quality score recalibration was performed using the Genome Analysis Toolkit (GATK)^75^ (v. 3.8.0). One subject’s samples did not pass quality control and were removed from further analysis, therefore this work utilized whole exome sequencing data for 71 subjects.

### Somatic variant analysis

Variant calling was performed using Mutect2 in tumor-normal mode^76^ following the best practices guidelines for exome-seq analysis provided by the GATK authors^77^. Variants were hard-filtered for quality, annotated with functional and consequence prediction using Ensembl’s Variant Effect Predictor^78^ (VEP v. 92) and converted to Mutation Annotation Format (MAF) using the vcf2maf tool (v. 1.6.16). MAF files for individual samples were concatenated into a combined MAF file spanning the full cohort for downstream analysis. The combined MAF file was used as an input for MutSigCV^22^ (v. 1.41) for driver gene analysis. MutSigCV relies on a background model that takes into account mutation abundance, clustering, and site conservation to identify genes that were mutated more often than expected by chance. A benchmarking study^79^ shows that this model imposes conservative selection criteria and may, therefore, fail to recognize candidate driver genes; moreover, the sample size required for near-comprehensive detection of intermediate-effect driver genes (90% detection and 2% effect size/increase with respect to background) was shown to be >300, i.e., several-fold larger than this study’s cohort size. Therefore, we expanded the driver gene selection criteria to also include frequently mutated genes (>10% of the samples in the Mongolian HCC cohort), although frequently mutated genes found in publicly available exome cohorts, termed FLAGS^80^, were excluded. Variants annotated with a frequency larger than 0.001 in the ExAC, gnomAD, or 1000 Genomes databases (i.e., common SNPs) were also removed. In addition, variants with less than 20x depth in the tumor sample and an alternate allele frequency of less than 5% were removed. Somatic mutation data for the TCGA-LIHC dataset were retrieved using the TCGAmutations (v. 0.2.0) R package, which provides pre-built objects using MAF files from the MC3 working group^81^. Visualization and summarization were performed using custom scripts in R (v. 3.6.0), primarily utilizing the packages maftools^82^ (v. 1.8.10) for data summarization, ComplexHeatmap^83^ (v. 2.1.0) and circlize^69^ (v. 0.4.8) for generating oncoplots, ribbon plots, and other circular plots, and trackViewer^84^ (v. 1.44.4) for lollipop plots.

### Copy number variant analysis

For each tumor-non-tumor pair, ploidy and purity estimates were computed with Sequenza^85^ (sequenza-utils v. 2.2 and sequenza R package v. 3.0), and these were used as inputs for CNV calling using the software package Control-Freec^86^ (v. 11.5). Regions with significant CNVs reported by Control-Freec were summarized for the cohort by first disjoining these regions into discrete non-overlapping segments. Next, segments were filtered for significance in each sample using the following criteria: Wilcoxon Rank Sum Test *p* value < 1e–3, Kolmogorov Smirnov *p* value < 1e–3, and Uncertainty between 0 and 20. Finally, the number of samples with CNVs in each segment were counted. Modified code from the R package svplucnv (v. 0.9.1) was used for summarization and visualization.

### Structural variant analysis

Structural variants (SVs) were called using Manta^87^ (v. 1.3.0) in paired tumor-non-tumor mode and annotated using AnnotSV^88^ (v. 1.1.1). These variants were filtered based on the sum of the split and spanning read counts for the mutant allele. SVs with more than 2 split or spanning reads in the nontumor sample, or less than 5 split or spanning reads in the tumor, were removed.

### Germline analysis

Germline variants were called using GATK’s HaplotypeCaller^89^ in joint genotyping mode. Variants were then filtered for quality with the following criteria: QD < 2.0, FS > 60.0, MQ < 40.0, MQRankSum < −12.5, ReadPosRankSum <−8.0 for SNPs; QD < 2.0, FS > 200.0, ReadPosRankSum < −20.0 for INDELs. Sample relatedness and ancestry were computed using the tool Somalier^90^ (v. 0.2.9), which is an updated implementation of Peddy^91^ that analyzes ancestry based on 17,766 common variants across all human populations. In order to visualize genetic similarity, pairwise relatedness values computed by Somalier were transformed as *value* -> 10 – 10^*value*, and then used for hierarchical clustering. In addition, genetic distance based on all germline variants found in the Mongolian HCC cohort was analyzed using PLINK^92^ (v. 1.9.0). To prioritize cancer-related germline variants, we utilized the Cancer Predisposition Sequencing Reporter^93^ (v. 0.5.1) to analyze 218 manually-curated cancer predisposition genes for known or predicted pathogenic variants.

### Mutational signature analysis

Trinucleotide frequency patterns were extracted with maftools^82^ (v. 1.8.10). Reference mutational signatures were obtained from the Catalogue Of Somatic Mutations In Cancer, versions v2 (March 2015)^30^ and v3 (May 2019)^31^, as well as from the Compendium of Mutational Signatures of Environmental Agents (May 2019)^33^. This information was fed into deconstructSigs^43^ (v. 1.8.0) to generate subject/signature weights from the non-negative least squares mapping of individual samples against the reference signatures. These weights are determined such that the reconstructed tumor sample matrix minimizes a given error threshold^43^. To reduce false positives, some corrections can be applied to the fitting approach; for example, deconstructSigs uses forward selection to estimate a minimal number of signatures and removes a signature’s contribution to a sample if it accounts for less than 6% of the sample’s mutations. Limitations of deconstructSigs and other mutational signature methods have been discussed and benchmarked elsewhere^94^. For each mutational signature compendium, the subject/signature weight matrix was obtained; then, signatures with distinct prevalence between HDV+ and HDV− groups were identified by the criterion of *p* < 0.05 in the Wilcoxon test performed between HDV+ and HDV− weight distributions. Only signatures that passed this selection criterion were selected (Fig. 4b).

### Mutated oncogenic signaling pathways

The online tool PathwayMapper^95^ (v. 2.0) was used to export as plain-text a set of ten pan-cancer oncogenic signaling pathway graphical templates derived from TCGA^54^. Each pathway plain-text template was then modified to contain only genes mutated in Mongolian HCC; for each of these genes, we provided the fraction of mutated samples in each molecular subclass MO1-4 and the sign of the median gene expression log ratio among mutated genes in each subclass. Pathways with none or few mutated genes were removed. Those remaining were individually uploaded into PathwayMapper to generate graphical renditions of mutated gene percentages and activation, exported as graphics, and then merged.

### Reporting summary

Further information on research design is available in the Nature Research Reporting Summary linked to this article.

## Supplementary information

Supplementary Information

Description of Additional Supplementary Files

Supplementary Data 1

Supplementary Data 2

Supplementary Data 3

Supplementary Data 4

Supplementary Data 5

Supplementary Data 6

Supplementary Data 7

Supplementary Data 8

Supplementary Data 9

Supplementary Data 10

Supplementary Data 11

Supplementary Data 12

Supplementary Data 13

Supplementary Data 14

Supplementary Data 15

Supplementary Data 16

Supplementary Data 17

Supplementary Data 18

Reporting Summary

## Data Availability

Public datasets used are TCGA (https://portal.gdc.cancer.gov) and MSigDB (https://www.gsea-msigdb.org/gsea/msigdb/index.jsp). Total-RNA Sequencing data are available at the Gene Expression Omnibus (GEO) repository under Study Accession GSE144269. Phenotypic and Whole-Exome Sequencing data are available at the dbGaP repository under Study Accession phs002000.v1.p1. Source data are provided with this paper. The remaining data are available in the Article, Supplementary Information, or available from the authors upon request. Source data are provided with this paper.

## Source data

Source Data

## Code availability

The scripts used in our bioinformatic pipeline to perform data analysis and visualization are available as a public GitHub repository at https://github.com/juliancandia/MongolianHCC. Source data are provided with this paper.
