# Supplementary material for: The genomic landscape of Mongolian hepatocellular carcinoma
Source: Nat Commun. 2020 Sep 1;11:4383. doi: 10.1038/s41467-020-18186-1 (PMC7462863; doi:10.1038/s41467-020-18186-1)
Supplement: Supplementary file 1 — Supplementary Information [file 41467_2020_18186_MOESM1_ESM.pdf]

Supplementary Figures

The genomic landscape of Mongolian hepatocellular carcinoma

Julián Candia[1,*], Enkhjargal Bayarsaikhan[2,*], Mayank Tandon[3,*], Anuradha Budhu[1,4], Marshonna Forgues[1], Lkhagva-Ochir Tovuu[2], Undarmaa Tudev[5], Justin Lack[3], Ann Chao[6], Jigjidsuren Chinburen[7], Xin Wei Wang[1,**]

[1]Laboratory of Human Carcinogenesis, Center for Cancer Research, National Cancer Institute, National Institutes of Health, Bethesda, MD 20892, USA. [2]General Laboratory Department, National Cancer Center, Ulaanbaatar, Mongolia. [3]CCR Collaborative Bioinformatics Resource, Center for Cancer Research, National Cancer Institute, National Institutes of Health, Bethesda, MD 20892, USA. [4]Liver Cancer Program, Center for Cancer Research, National Cancer Institute, National Institutes of Health, Bethesda, MD 20892, USA. [5]Cancer Registry and Screening Department, National Cancer Center, Ulaanbaatar, Mongolia. [6]Center for Global Health, National Cancer Institute, National Institutes of Health, Rockville, MD 20850, USA. [7]Hepato-Pancreatic-Biliary Surgical Department, National Cancer Center, Ulaanbaatar, Mongolia.

[*]Contributed equally to this work.

[**]Correspondence: xw3u@nih.gov (X.W.W.)

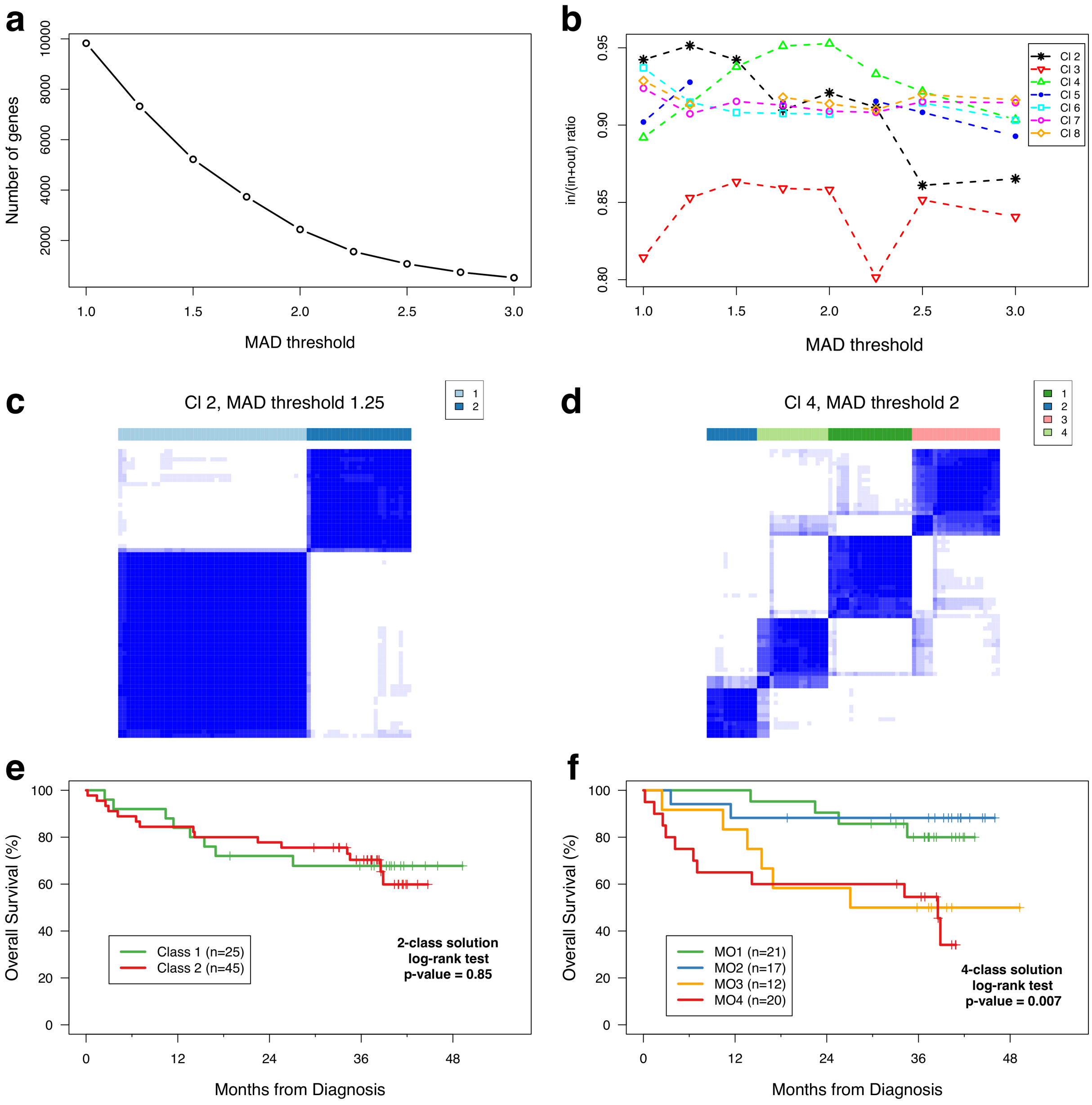

Supplementary Figure 1: Consensus clustering uncovers transcriptome-based molecular subclasses. (a) Number of genes selected as a function of the median absolute deviation (MAD) threshold. **(b)** Mean co-clustering ratio vs MAD threshold (in = mean pairwise co-clustering within a cluster, out = mean pairwise co-clustering across clusters), which is used as objective function to find the optimal solution. **(c)** Co-clustering matrix[1] for a solution with 2 clusters and MAD threshold=1.25. **(d)** Co-clustering matrix for a solution with 4 clusters and MAD threshold=2. **(e)**

Kaplan-Meier survival plots for the 2-cluster solution. The two-sided log-rank test yields p=0.85. **(f)** Kaplan-Meier survival plots for the 4-cluster solution. The two-sided log-rank test yields p=0.007. This is the solution adopted for downstream analysis. **Fig. 1(b)** (in the main text) also reports log-rank test p-values for the comparison between better prognosis (MO1-2) vs poorer prognosis (MO3-4) molecular subclasses, both individually and grouped pairwise.

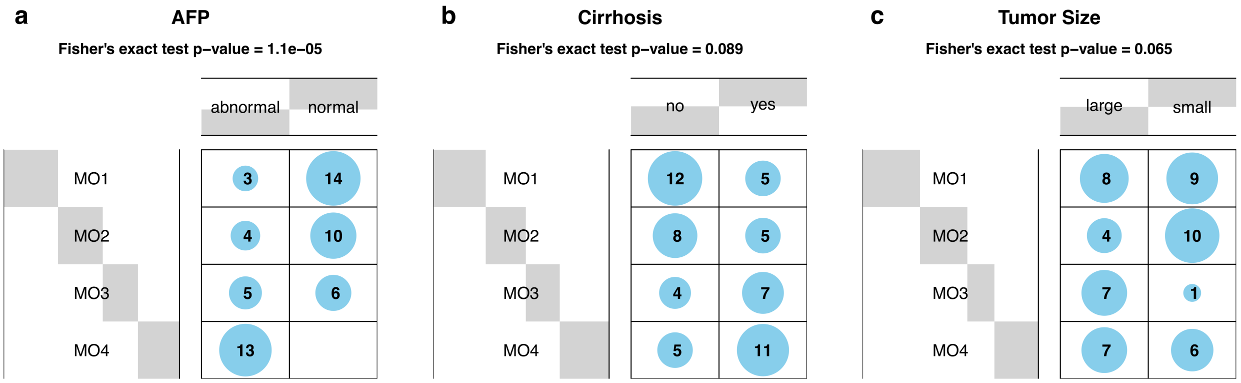

Supplementary Figure 2: Association between molecular subclasses MO1-4 and clinical variables. Contingency tables and two-sided Fisher's exact test p-values are shown for **(a)** alpha-fetoprotein (AFP), **(b)** cirrhosis, and **(c)** tumor size.

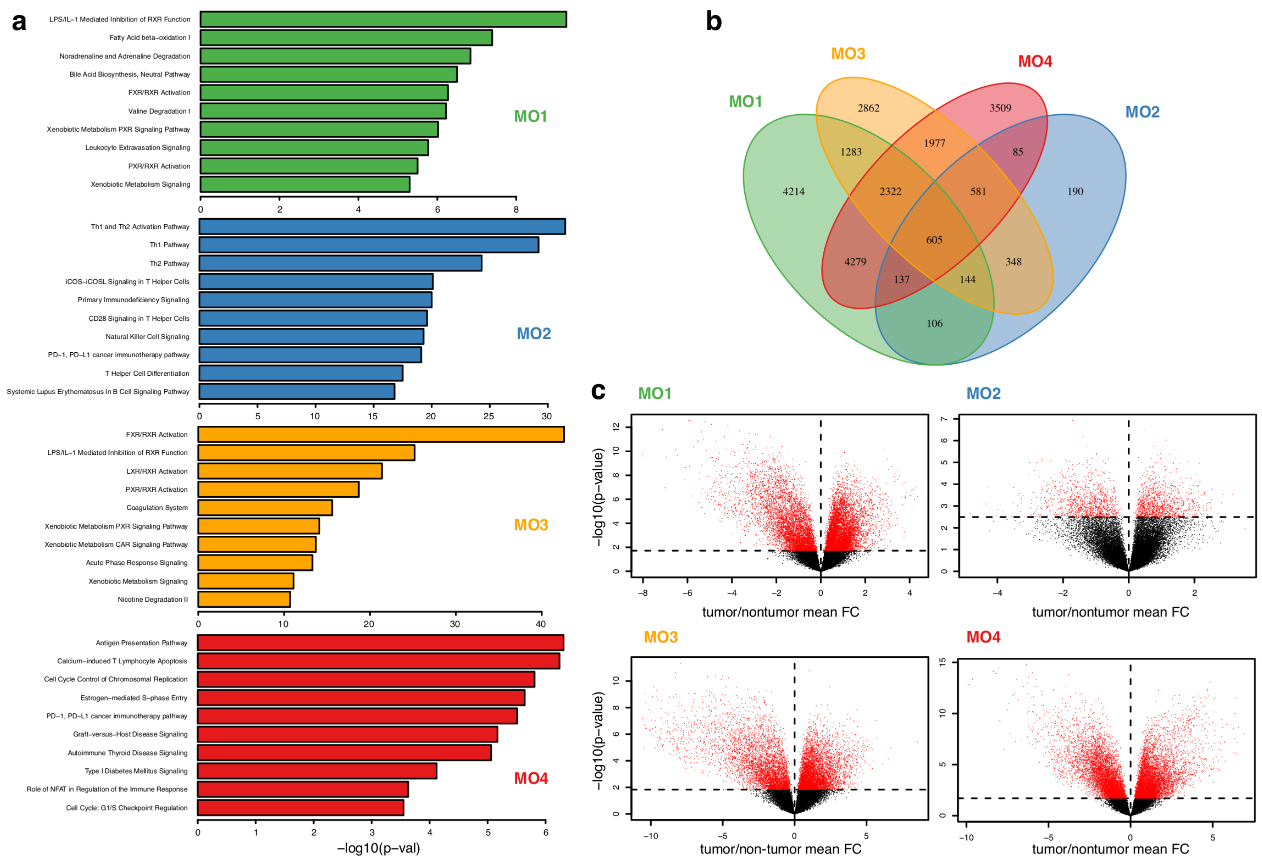

Supplementary Figure 3: Differentially expressed genes and pathways for each molecular subclass in Mongolian HCC. (a) Ingenuity Pathway Analysis generated from differentially expressed genes in each molecular subclass. Full analysis results are provided in **Supplementary Data 4. (b)** Venn diagram of tumor-vs-nontumor differentially expressed genes in each molecular subclass (gene lists provided in **Supplementary Data 5**). **(c)** Volcano plots of tumor-vs-nontumor differentially expressed genes in each molecular subclass. The vertical axes show -$\log_{10}$(p-value), where p-values were determined from two-sided paired t-tests without multiple-testing correction. In red, genes differentially expressed with FDR-adjusted p-value<0.05. Detailed numerical results provided in **Supplementary Data 5**.

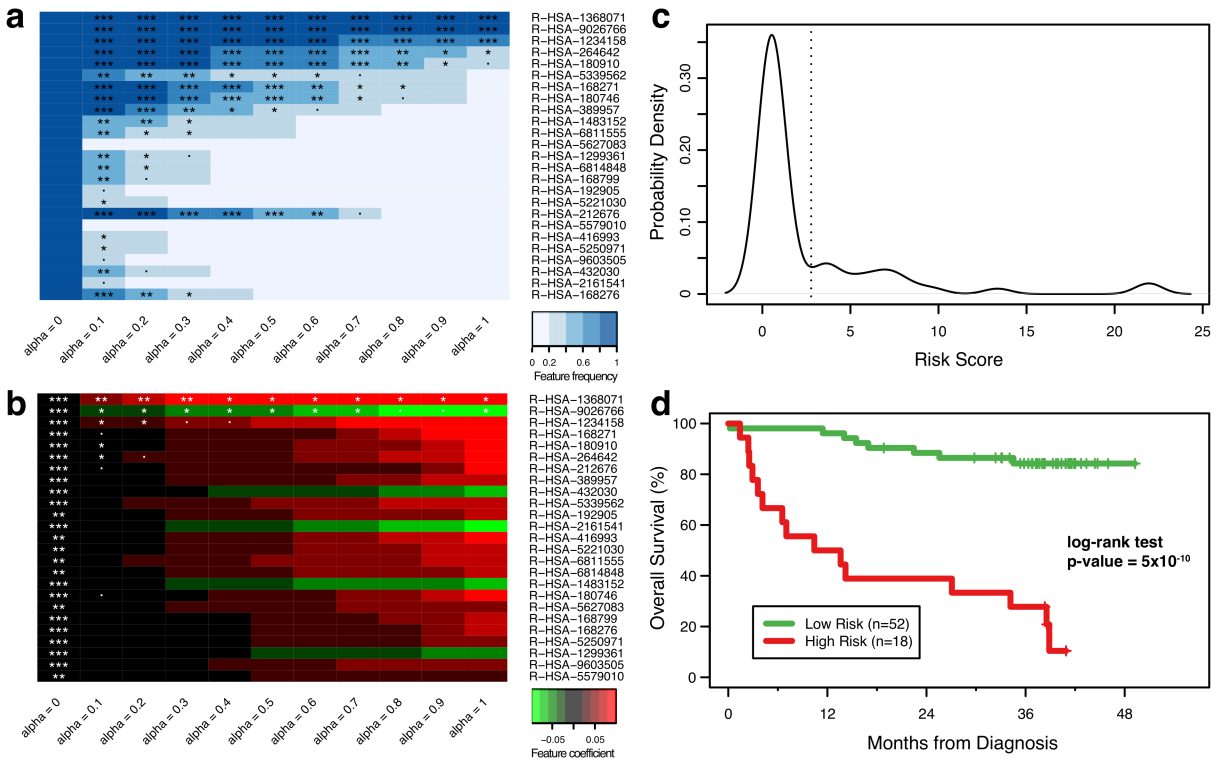

Supplementary Figure 4: Supervised analysis (based on regularized Cox regression[2]) uncovers low- vs high-risk groups. (a) Reactome pathways most predictive of survival according to the frequency of selection in cross-validated Cox regression models. A family of regularized elastic net models from ridge (α=0) to lasso (α=1) is shown; features appear ordered in decreasing order of significance based on the most stringent (lasso) solution. P-value significance codes: <0.001 (***), <0.01 (**), <0.05 (*), <0.1(*). Full analysis results are provided in **Supplementary Data 6. (b)** Pathways selected in (a), here shown based on their Cox regression coefficients; negative coefficients (green) indicate correlation with lower risk, while positive coefficients (red) are associated with higher risk. **(c)** Probability density of risk scores distributed across the cohort. The dashed line separates low- vs high-risk patients. **(d)** Overall survival for subjects stratified in low- vs high-risk groups (two-sided log-rank test p-value=$5x10^{-10}$).

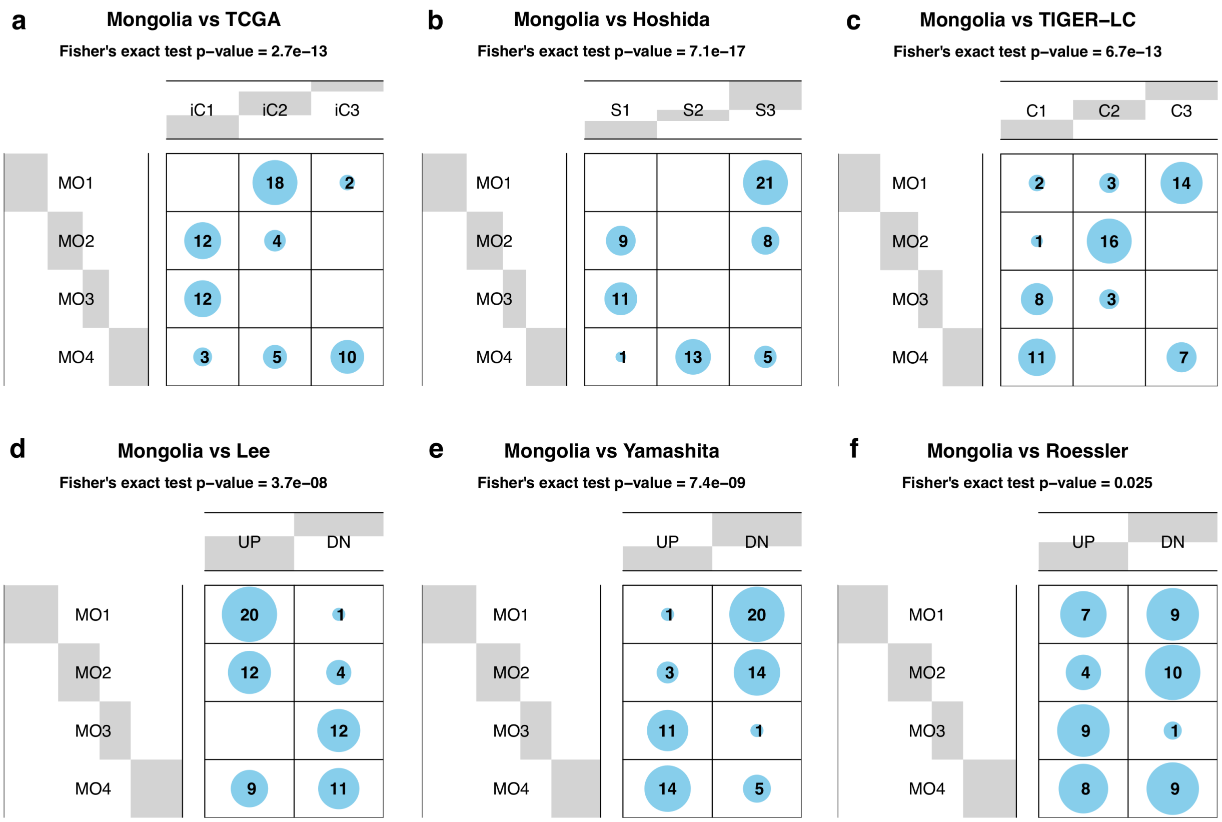

Supplementary Figure 5: Comparison between molecular subclass gene signatures from different HCC studies. Using the Nearest Template Prediction method[3], the Mongolian HCC cohort was classified according to molecular subclass schemes reported in previous studies. This figure shows contingency tables and two-sided Fisher's exact test p-values for the comparison between the molecular subclasses MO1-4 (this study) and those from **(a)** TCGA[4], **(b)** Hoshida[5], **(c)** TIGER-LC[6], **(d)** Lee[7], **(e)** Yamashita[8], and **(f)** Roessler[9].

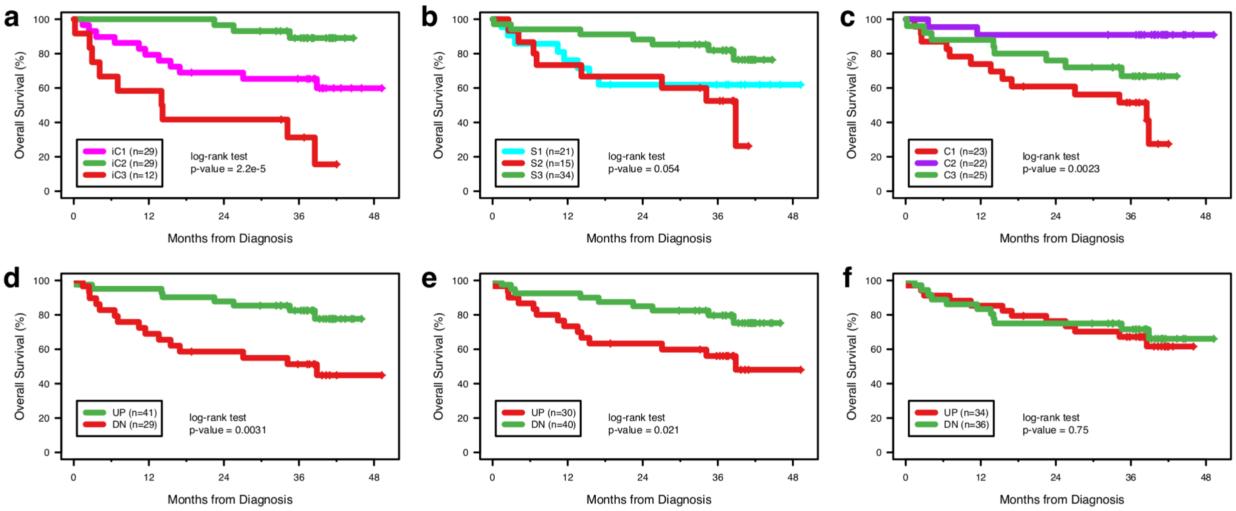

Supplementary Figure 6: Overall survival for Mongolian HCC subjects stratified according to gene signatures from other HCC studies: **(a)** TCGA[4], **(b)** Hoshida[5], **(c)** TIGER-LC[6], **(d)** Lee[7], **(e)** Yamashita[8], and **(f)** Roessler[9]. In all cases, the statistical significance of survival stratification was assessed via two-sided log-rank test p-value.

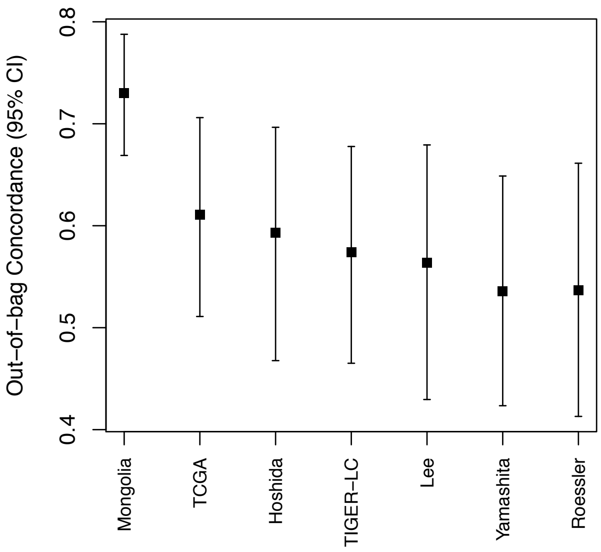

Supplementary Figure 7: Comparative performance of cross-validated Mongolian HCC survival prediction using gene signatures from this study (Mongolia) and those from other HCC studies, namely TCGA[4], Hoshida[5], TIGER-LC[6], Lee[7], Yamashita[8], and Roessler[9]. The error bars show the 95% CI out-of-bag concordance determined from 200 independent realizations of 10-fold cross-validated runs.

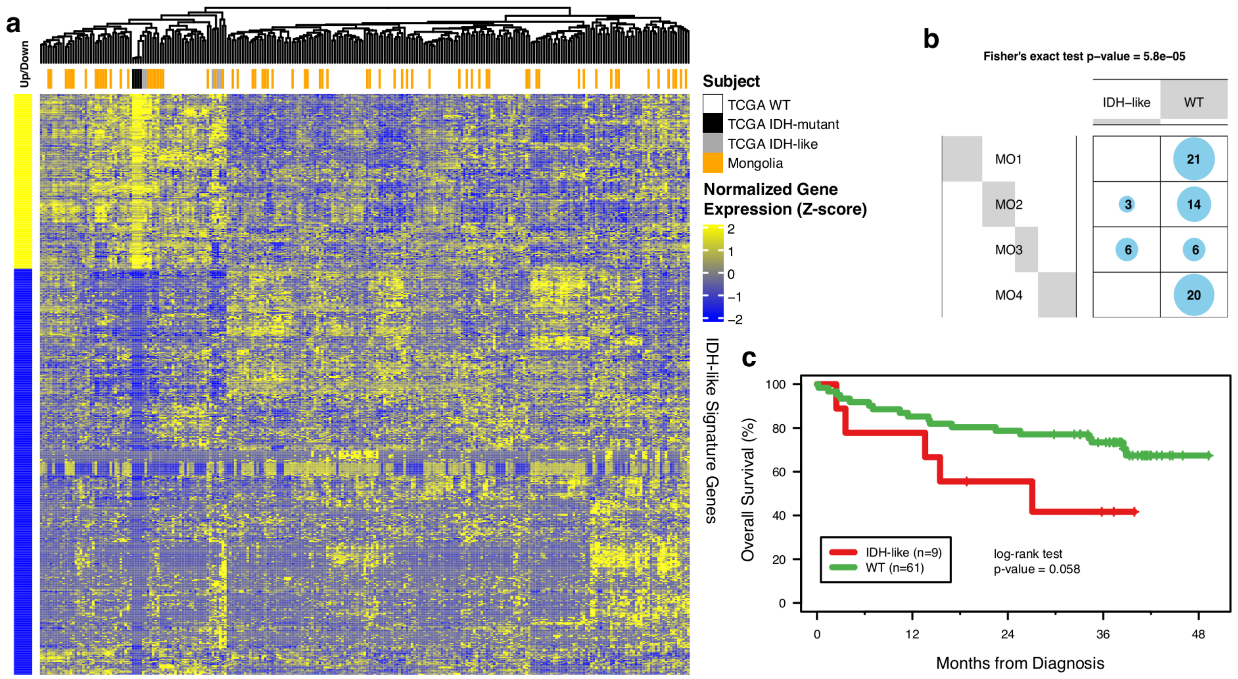

Supplementary Figure 8: IDH-like phenotype in Mongolian HCC. (a) Heatmap of hierarchically clustered TCGA and Mongolian HCC samples (columns) vs IDH-like signature genes (rows). IDH-mutant (black) and IDH-like (grey) samples from TCGA appear annotated on the bar on top, as well as all Mongolian (orange) samples. Seven of them (Patient Ids 7, 27, 40, 62, 71, 74, 78) form a cluster adjacent to the IDH-mutant TCGA samples; other two (Patient Ids 13, 67) form a separate cluster adjacent to some IDH-like TCGA samples. **(b)** Association between IDH-like status and molecular subclasses in Mongolian HCC assessed via two-sided Fisher's exact test. **(c)** Overall survival for Mongolian HCC subjects stratified by the IDH-like signature assessed via two-sided log-rank test.

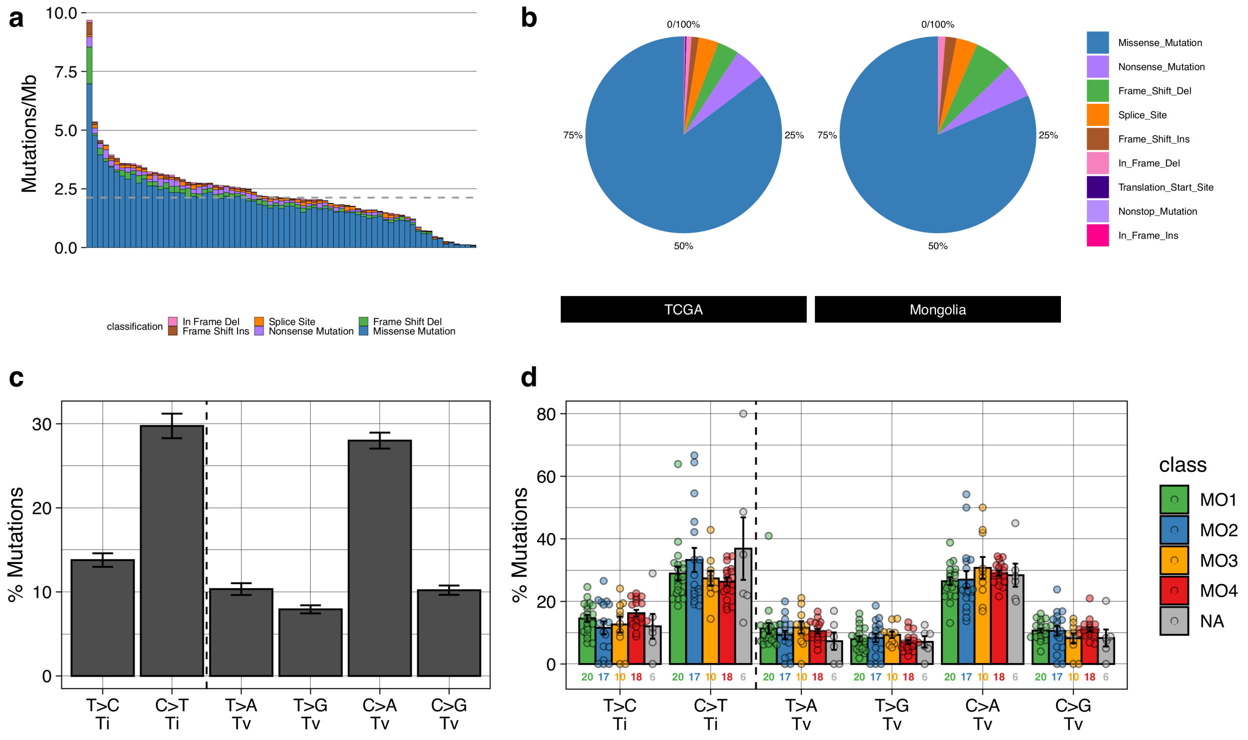

Supplementary Figure 9: Mutation burden statistics in the Mongolian HCC cohort. (a) Average number of mutations per mega-base per sample. Median=2.12 (dashed line), Q1=1.57, Q3=2.85. **(b)** Frequency distribution of genomic alterations by type (right) compared to TCGA-LIHC (left). **(c)** Frequency distribution of single-base substitutions by type (Ti=transition, Tv=transversion). Error bars represent the mean fraction of variants ± SEM (n=71 biologically independent samples). **(d)** Frequency distribution of single-base substitutions by type and molecular subclass. Error bars represent the mean fraction of variants ± SEM. The number of biologically independent samples in each group is indicated below each bar.

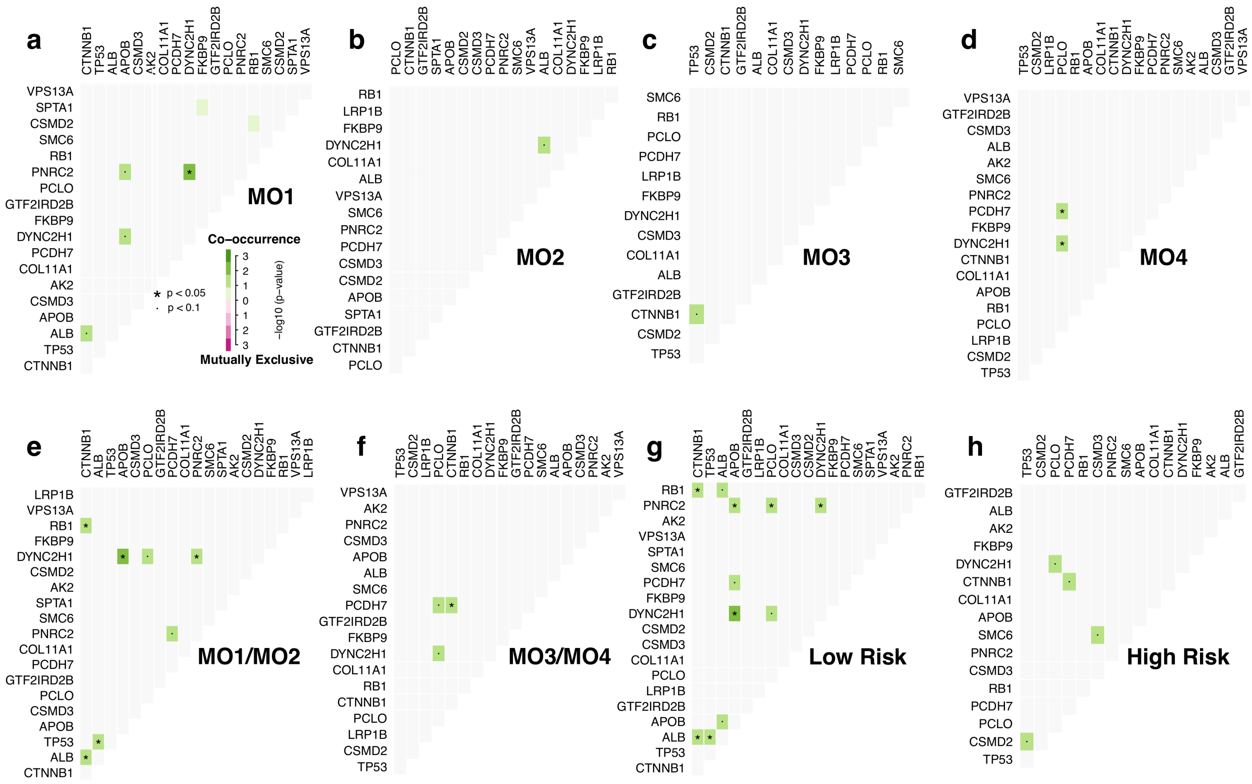

Supplementary Figure 10: Pairwise association between driver gene mutations in Mongolian HCC sub-cohorts. Genes that were not mutated in a given sub-cohort are not shown. **(a-d)** Individual molecular subclasses MO1-4. **(e)** Better-prognosis molecular subclasses MO1-2. **(f)** Worse-prognosis molecular subclasses MO3-4. **(g)** Low risk subgroup (based on supervised transcriptome analysis). **(h)** High risk subgroup (based on supervised transcriptome analysis). For all analyses, p-values were determined from two-sided Fisher's exact tests without multiple-testing correction. Detailed numerical results are provided in **Supplementary Data 12**.

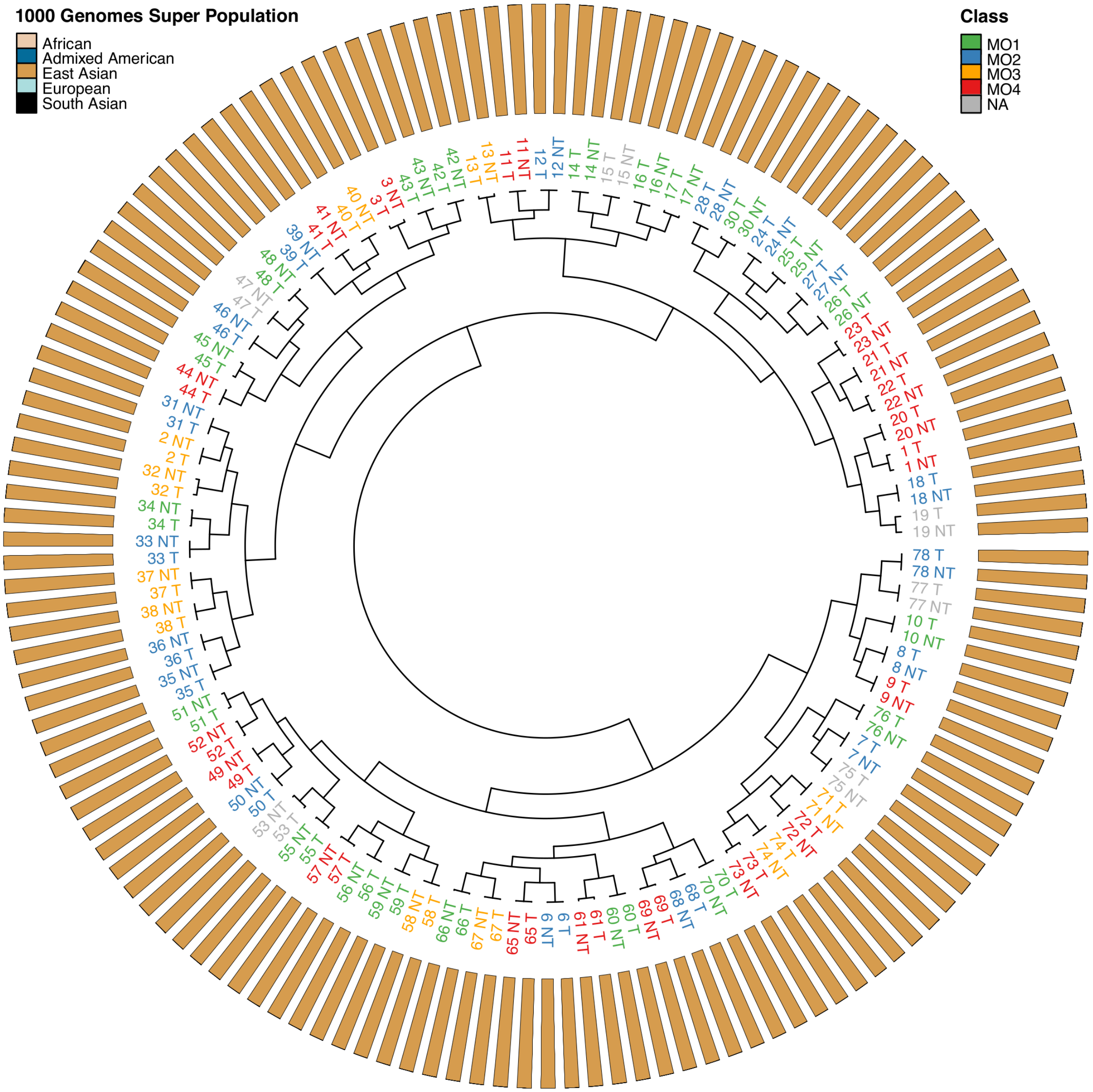

Supplementary Figure 11: Germline-based ancestry admixture and sample similarity. Sample relatedness for tumor (T) and adjacent non-tumor (NT) samples is indicated by the circular dendrogram obtained via hierarchical clustering. Ancestry based on the 1000 Genomes Super Populations is shown in the outer ring. Sample labels are colored according to molecular subclass.

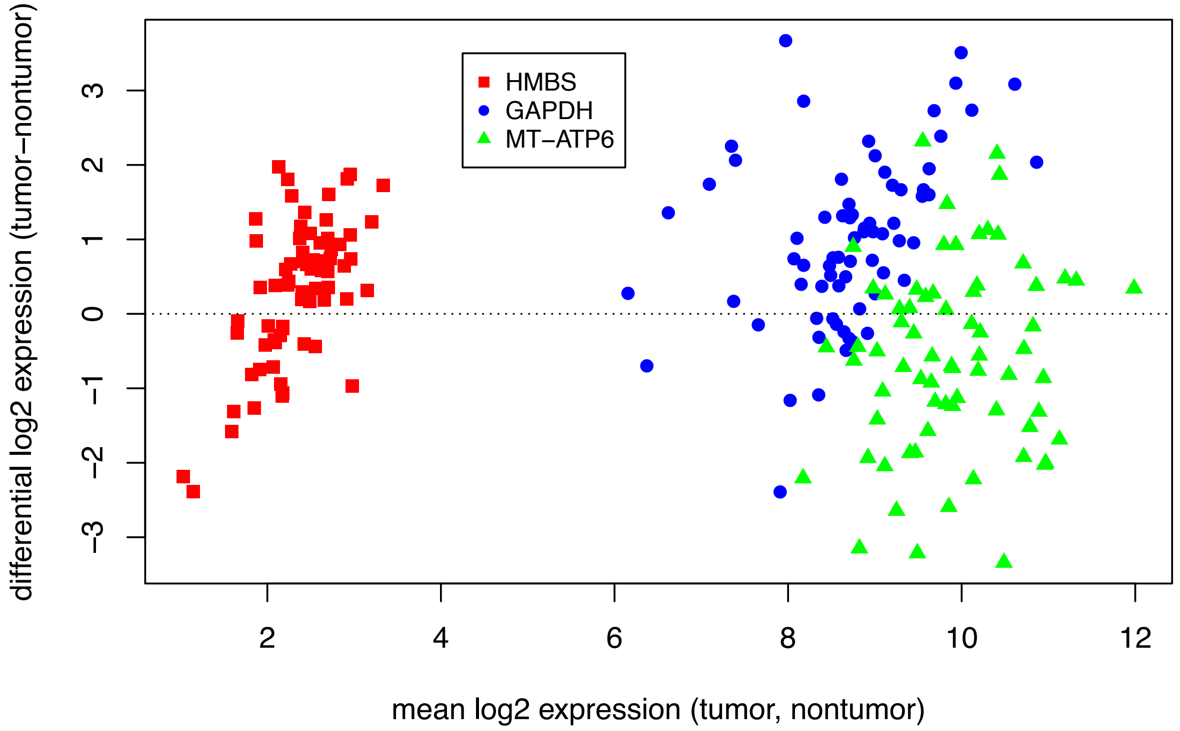

Supplementary Figure 12: Bland-Altman plot showing the expression of reference housekeeping genes reported as stable across tumor and normal tissues[10-12]. Symbols represent individual patients.

Supplementary References

1. Wilkerson, M.D. & Hayes, D.N. ConsensusClusterPlus: a class discovery tool with confidence assessments and item tracking. *Bioinformatics* **26**, 1572-3 (2010).
2. Candia, J. & Tsang, J.S. eNetXplorer: an R package for the quantitative exploration of elastic net families for generalized linear models. *BMC bioinformatics* **20**, 189 (2019).
3. Hoshida, Y. Nearest template prediction: a single-sample-based flexible class prediction with confidence assessment. *PLoS One* **5**, e15543 (2010).
4. TheCancerGenomeAtlasResearchNetwork. Comprehensive and Integrative Genomic Characterization of Hepatocellular Carcinoma. *Cell* **169**, 1327-1341 e23 (2017).
5. Hoshida, Y. *et al.* Integrative transcriptome analysis reveals common molecular subclasses of human hepatocellular carcinoma. *Cancer Res* **69**, 7385-92 (2009).
6. Chaisaingmongkol, J. *et al.* Common Molecular Subtypes Among Asian Hepatocellular Carcinoma and Cholangiocarcinoma. *Cancer Cell* **32**, 57-70 e3 (2017).
7. Lee, J.S. *et al.* Classification and prediction of survival in hepatocellular carcinoma by gene expression profiling. *Hepatology* **40**, 667-676 (2004).
8. Yamashita, T. *et al.* EpCAM and alpha-fetoprotein expression defines novel prognostic subtypes of hepatocellular carcinoma. *Cancer Res.* **68**, 1451-1461 (2008).
9. Roessler, S. *et al.* A unique metastasis gene signature enables prediction of tumor relapse in early-stage hepatocellular carcinoma patients. *Cancer Research* **70**, 10202-10212 (2010).
10. Gerard, C.J., Andrejka, L.M. & Macina, R.A. Mitochondrial ATP synthase 6 as an endogenous control in the quantitative RT-PCR analysis of clinical cancer samples. *Mol Diagn* **5**, 39-46 (2000).
11. Janssens, N., Janicot, M., Perera, T. & Bakker, A. Housekeeping genes as internal standards in cancer research. *Mol Diagn* **8**, 107-13 (2004).
12. Cicinnati, V.R. *et al.* Validation of putative reference genes for gene expression studies in human hepatocellular carcinoma using real-time quantitative RT-PCR. *BMC Cancer* **8**, 350 (2008).
