# Supplementary material for: The genomic landscape of Mongolian hepatocellular carcinoma
Source: Nat Commun. 2020 Sep 1;11:4383. doi: 10.1038/s41467-020-18186-1 (PMC7462863; doi:10.1038/s41467-020-18186-1)
Supplement: Supplementary file 2 — Description of Additional Supplementary Files [file 41467_2020_18186_MOESM2_ESM.pdf]

Description of Additional Supplementary Files

File Name: Supplementary Data 1
Description: Demographic, clinical, and laboratory characteristics of patients at time of surgery.

File Name: Supplementary Data 2
Description: Patient-level demographic and clinical information.

File Name: Supplementary Data 3
Description: Differentially expressed genes characteristic of molecular subclasses MO1-4.

File Name: Supplementary Data 4
Description: Pathways from differentially expressed genes in molecular subclasses MO1-4.

File Name: Supplementary Data 5
Description: Tumor vs non-tumor mean fold-change, two-sided paired t-test p-value, and FDR-adjusted p-value for each molecular subclass in Mongolian HCC.

File Name: Supplementary Data 6
Description: Feature- and coefficient-based p-values across the family of regularized Cox regression models from ridge (alpha=0) to lasso (alpha=1) obtained by the R package eNetXplorer.

File Name: Supplementary Data 7
Description: Differentially expressed genes characteristic of each one of the molecular subclasses from previous HCC studies (TCGA, Hoshida, TIGER-LC, Lee, Yamashita, and Roessler).

File Name: Supplementary Data 8
Description: Candidate driver gene analysis for Mongolian HCC based on MutSigCV and mutation frequencies.

File Name: Supplementary Data 9
Description: Rank-ordered contributions of somatic substitution patterns to PC1, PC2, and PC3.

File Name: Supplementary Data 10
Description: Driver genes identified in the Mongolian cohort, compared with previous HCC and pan-cancer studies.

File Name: Supplementary Data 11
Description: Driver genes not found in the Mongolian cohort, compared with previous HCC studies.

File Name: Supplementary Data 12
Description: Co-occurrence of mutated driver genes in Mongolian HCC (full cohort and subcohorts).

File Name: Supplementary Data 13
Description: Mutated loci in all reported candidate driver genes for Mongolian HCC.

File Name: Supplementary Data 14
Description: Frequency distributions of single-nucleotide substitutions in the Mongolian HCC cohort and HDV+/- subcohorts.

File Name: Supplementary Data 15
Description: Gene fusions in Mongolian HCC.

File Name: Supplementary Data 16
Description: Copy-number variations in Mongolian HCC.

File Name: Supplementary Data 17
Description: Structural variants in Mongolian HCC.

File Name: Supplementary Data 18
Description: Germline variant analysis in Mongolian HCC.
