# Supplementary material for: The genomic landscape of Mongolian hepatocellular carcinoma
Source: Nat Commun. 2020 Sep 1;11:4383. doi: 10.1038/s41467-020-18186-1 (PMC7462863; doi:10.1038/s41467-020-18186-1)
Supplement: Supplementary file 21 — Reporting Summary [file 41467_2020_18186_MOESM21_ESM.pdf]

nature research

Corresponding author(s): Xin Wei Wang
Last updated by author(s): Jul 20, 2020

Reporting Summary

Nature Research wishes to improve the reproducibility of the work that we publish. This form provides structure for consistency and transparency in reporting. For further information on Nature Research policies, see our Editorial Policies and the Editorial Policy Checklist.

Statistics

For all statistical analyses, confirm that the following items are present in the figure legend, table legend, main text, or Methods section.

n/a	Confirmed	
☐	☒	The exact sample size (*n*) for each experimental group/condition, given as a discrete number and unit of measurement
☐	☒	A statement on whether measurements were taken from distinct samples or whether the same sample was measured repeatedly
☐	☒	The statistical test(s) used AND whether they are one- or two-sided<br>*Only common tests should be described solely by name; describe more complex techniques in the Methods section.*
☐	☒	A description of all covariates tested
☐	☒	A description of any assumptions or corrections, such as tests of normality and adjustment for multiple comparisons
☐	☒	A full description of the statistical parameters including central tendency (e.g. means) or other basic estimates (e.g. regression coefficient) AND variation (e.g. standard deviation) or associated estimates of uncertainty (e.g. confidence intervals)
☐	☒	For null hypothesis testing, the test statistic (e.g. *F*, *t*, *r*) with confidence intervals, effect sizes, degrees of freedom and *P* value noted<br>*Give P values as exact values whenever suitable.*
☒	☐	For Bayesian analysis, information on the choice of priors and Markov chain Monte Carlo settings
☒	☐	For hierarchical and complex designs, identification of the appropriate level for tests and full reporting of outcomes
☒	☐	Estimates of effect sizes (e.g. Cohen's *d*, Pearson's *r*), indicating how they were calculated

Our web collection on statistics for biologists contains articles on many of the points above.

Software and code

Policy information about availability of computer code

Data collection	No software was used.
Data analysis	Software used: R (v. 3.6.0), FastQC (v. 0.11.5), Preseq (v. 2.0.3), Picard tools (v. 1.119), RSeQC (v. 2.6.4), Cutadapt (v. 1.14), STAR (v. 2.5.2b), RSEM (v. 1.3.0), Limma (v. 3.40.6), ConsensusClusterPlus (v. 1.48.0), QIAGEN Ingenuity Pathway Analysis (v. 52912811), VennDiagram (v. 1.6.20), eNetXplorer (v. 1.1.0), GenePattern's NearestTemplatePrediction module (v. 4), circlize (v. 0.4.8), survival (v. 3.1.8), survcomp (v. 1.34.0), glmnet (v. 3.0.1), STAR-fusion (v. 1.6), BWA (v. 0.7.17), GATK (v. 3.8.0), VEP (v. 92), vcf2maf (v. 1.6.16), MutSigCV (v. 1.41), TCGAmutations (v. 0.2.0), maftools (v. 1.8.10), ComplexHeatmap (v. 2.1.0), trackViewer (v. 1.44.4), sequenza-utils (v. 2.2), sequenza R package (v. 3.0), Control-Freec (v. 11.5), svplucnv (v. 0.9.1), Manta (v. 1.3.0), AnnotSV (v. 1.1.1), Somalier (v. 0.2.9), PLINK (v. 1.9.0), Cancer Predisposition Sequencing Reporter (v. 0.5.1), deconstructSigs (v. 1.8.0), PathwayMapper (v. 2.0). Custom software is available at https://github.com/juliancandia/MongolianHCC

For manuscripts utilizing custom algorithms or software that are central to the research but not yet described in published literature, software must be made available to editors and reviewers. We strongly encourage code deposition in a community repository (e.g. GitHub). See the Nature Research guidelines for submitting code & software for further information.

Data

Policy information about availability of data

All manuscripts must include a data availability statement. This statement should provide the following information, where applicable:

- Accession codes, unique identifiers, or web links for publicly available datasets
- A list of figures that have associated raw data
- A description of any restrictions on data availability

Public datasets used are TCGA (https://portal.gdc.cancer.gov) and MSigDB (https://www.gsea-msigdb.org/gsea/msigdb/index.jsp). Total-RNA Sequencing data are

available at the Gene Expression Omnibus (GEO) repository under Study Accession GSE144269. Phenotypic and Whole-Exome Sequencing data are available at the dbGaP repository under Study Accession phs002000.v1.p1

Field-specific reporting

Please select the one below that is the best fit for your research. If you are not sure, read the appropriate sections before making your selection.

☒ Life sciences ☐ Behavioural & social sciences ☐ Ecological, evolutionary & environmental sciences

For a reference copy of the document with all sections, see nature.com/documents/nr-reporting-summary-flat.pdf

Life sciences study design

All studies must disclose on these points even when the disclosure is negative.

Sample size	Sample size was determined by the available tissue collection with informed consent of hepatocellular carcinoma (HCC) patients undergoing surgery between 2015 and 2016 at the National Cancer Center in Mongolia. Clinical and demographic characteristics of the cohort were deemed representative of the Mongolian HCC population.
Data exclusions	As stated in the Methods section, the whole-exome sequencing tumor sample for one patient did not pass quality control and was excluded from the study. Exclusion criteria were pre-established by standard QC criteria built into our whole-exome sequencing pipeline.
Replication	No replication was made due to limitations imposed by the sample collection process.
Randomization	Not relevant to our study because there was no experimental group allocation.
Blinding	Blinding was not relevant, since there were no expected outcomes or pre-defined outcome measures. This is a quantitative, descriptive study of the genomic landscape of Mongolian HCC.

Reporting for specific materials, systems and methods

We require information from authors about some types of materials, experimental systems and methods used in many studies. Here, indicate whether each material, system or method listed is relevant to your study. If you are not sure if a list item applies to your research, read the appropriate section before selecting a response.

Materials & experimental systems

n/a	Involved in the study	
☒	☐	Antibodies
☒	☐	Eukaryotic cell lines
☒	☐	Palaeontology and archaeology
☒	☐	Animals and other organisms
☐	☒	Human research participants
☒	☐	Clinical data
☒	☐	Dual use research of concern

Methods

n/a	Involved in the study	
☒	☐	ChIP-seq
☒	☐	Flow cytometry
☒	☐	MRI-based neuroimaging

Human research participants

Policy information about studies involving human research participants

Population characteristics	76 HCC patients. 37 (49%) female, 38 (51%) male. Ages between 23 and 77 (median age: 60). 43 (57%) infected with Hepatitis Virus HCV. 53 (70%) infected with Hepatitis Virus HBV. 28 (37%) infected with Hepatitis Virus HDV (all of them also infected with HBV). Germline-based ancestry admixture analysis showed all patients >99% East Asian based on the 1000 Genomes Super Populations (result included in the manuscript). These characteristics were deemed representative of the Mongolian HCC population.
Recruitment	HCC patients undergoing surgery at the National Cancer Center, Ulaanbaatar, Mongolia. The cohort is considered representative of the Mongolian population. There are no known potential biases that may affect the results.
Ethics oversight	Ethics Committee at the National Cancer Center in Ulaanbaatar, Mongolia.

Note that full information on the approval of the study protocol must also be provided in the manuscript.
